# The influence of parental practices on child promotive and preventive food consumption behaviors: a systematic review and meta-analysis

**DOI:** 10.1186/s12966-017-0501-3

**Published:** 2017-04-11

**Authors:** Andrew Z. H. Yee, May O. Lwin, Shirley S. Ho

**Affiliations:** Wee Kim Wee School of Communication and Information, Nanyang Technological University, 31 Nanyang Link, Singapore, Singapore 637718

**Keywords:** Parent, Child, Nutrition, Food, Eating, Fruits, Vegetables, Sugar, Healthy, Unhealthy, Consumption

## Abstract

**Background:**

The family is an important social context where children learn and adopt eating behaviors. Specifically, parents play the role of health promoters, role models, and educators in the lives of children, influencing their food cognitions and choices. This study attempts to systematically review empirical studies examining the influence of parents on child food consumption behavior in two contexts: one promotive in nature (e.g., healthy food), and the other preventive in nature (e.g., unhealthy food).

**Methods:**

From a total of 6,448 titles extracted from Web of Science, ERIC, PsycINFO and PubMED, seventy eight studies met the inclusion criteria for a systematic review, while thirty seven articles contained requisite statistical information for meta-analysis. The parental variables extracted include active guidance/education, restrictive guidance/rule-making, availability, accessibility, modeling, pressure to eat, rewarding food consumption, rewarding with verbal praise, and using food as reward. The food consumption behaviors examined include fruits and vegetables consumption, sugar-sweetened beverages, and snack consumption.

**Results:**

Results indicate that availability (Healthy: *r* = .24, *p* < .001; Unhealthy: *r* = .34, *p* < .001) and parental modeling effects (Healthy: *r* = .32, *p* < .001; Unhealthy: *r* = .35, *p* < .001) show the strongest associations with both healthy and unhealthy food consumption. In addition, the efficacy of some parenting practices might be dependent on the food consumption context and the age of the child. For healthy foods, active guidance/education might be more effective (*r* = .15, *p* < .001). For unhealthy foods, restrictive guidance/rule-making might be more effective (*r* = −.11, *p* < .01). For children 7 and older, restrictive guidance/rule-making could be more effective in preventing unhealthy eating (*r* = − .20, *p* < .05). For children 6 and younger, rewarding with verbal praise can be more effective in promoting healthy eating (*r* = .26, *p* < .001) and in preventing unhealthy eating (*r* = − .08, *p* < .01).

**Conclusions:**

This study illustrates that a number of parental behaviors are strong correlates of child food consumption behavior. More importantly, this study highlights 3 main areas in parental influence of child food consumption that are understudied: (1) active guidance/education, (2) psychosocial mediators, and (3) moderating influence of general parenting styles.

**Electronic supplementary material:**

The online version of this article (doi:10.1186/s12966-017-0501-3) contains supplementary material, which is available to authorized users.

## Background

Food consumption preferences are developed early in life [1]. Understanding how children’s food consumption choices are developed has the potential to benefit individuals’ health over their entire lifetime. Specifically, limiting the consumption of sugar-sweetened beverages (SSBs), while increasing the consumption of healthy food choices such as fruits and vegetables, can have protective effects on people’s health [2, 3]. In spite of this, children across several parts of the world are consuming sugars at an alarming rate, with children in the United States [4, 5], United Kingdom [6], Mexico [7], and even Asian countries such as Taiwan and Singapore [8–10] consuming SSBs at worrying levels. To compound the problem of sugar consumption on their diets, the consumption of fruits and vegetables among children is relatively low across the world. In the United States, 60% of children do not consume enough fruits to meet the recommended daily guidelines, while 93% of children do not consume sufficient vegetables [11]. Mirroring the United States, European children are consuming fruits and vegetables below the recommended levels [12].

Parents are important socialization agents who play the role of health promoters, role models, and educators in the lives of their children [13]. Defined as “*processes whereby naïve individuals are taught the skills, behavior patterns, values, and motivations needed for competent functioning* (p.13)”, socialization in the context of food consumption involves parents conveying learning outcomes such as norms, knowledge, attitudes and behaviors to children via a range of behaviors [14]. Among socialization researchers, two broad concepts have been used to understand parental influence on child outcomes [15]. First, parental practices are context-specific strategies parents use to help children achieve socialization goals. Second, general parenting style, which cuts across behavioral contexts, refers to the general emotional climate in which these parental practices are situated.

Currently, there are no comprehensive systematic reviews surveying the influence of parental practices and parenting styles on child SSBs and fruits and vegetables consumption. Existing reviews have either focused on examining a broad array of determinants of SSBs consumption without an in-depth examination of the role of parents [16, 17], or examined the influence of singular parental practices – such as availability and role modelling – on various child food consumption behavior [18, 19]. Reviews that have focused on parental influence have examined parental practices in only one specific food consumption outcome, namely fruits and vegetables consumption, and these reviews tend not to quantitatively summarize the effect sizes between the parenting variables and child food consumption outcomes [20, 21]. There are currently no meta-analytic studies that aim to provide a quantitative summary of the influence of various parenting variables on child food consumption behavior. To our knowledge, this study would be the first to systematically review the influence of parents in two areas of child food consumption, one promotive (fruits and vegetables consumption) and one preventive (SSBs consumption). Examining the influence of parents in these two areas of child food consumption behaviors can help provide greater clarity of the efficacy of certain parental practices in different contexts.

This paper addresses these gaps in the literature by presenting the results from a comprehensive systematic review and meta-analysis of studies designed to examine the relationship between parental factors (which includes both *context-specific parental practices* and more *general parenting styles*) and child food consumption behaviors (SSBs and fruits and vegetables consumption). It attempts to cover a wide spectrum of parental factors believed to shape child food consumption, and examine their influence in two food consumption contexts – one preventive in nature (SSBs consumption) and one promotive in nature (fruits and vegetables consumption).

## Methods

The review was conducted in two phases. First, an extensive systematic review of the literature was done to identify the parental factors related to child food consumption behavior. Next, a meta-analysis was conducted following these classifications, with studies which contain the requisite statistical information.

### Literature search

In order to find relevant studies of parenting effects on child food cognitions, choice, and intake, a literature search was conducted in the following major databases: Web of Science, PsycINFO, ERIC, and PubMED. The search strategy relied on using a combination of parental factors keywords and child food consumption keywords. Parental factors keywords were matched with a child food consumption keyword for every search. The parental factor keywords were: “parenting”, “parent* communication”, “parent* strateg*”, “parent* style*”, “parent* practice*”, “parent* feed* strateg*”, and “parent* feed* practice*”. The keywords for child food consumption were: “eating”, “food consumption”, “soft drink*”, “sweetened beverage*”, “fruit*”, and “vegetable*”. To ensure comprehensiveness, the reference lists of key articles were hand searched to identify literature not retrieved from the initial search. An initial search was conducted in December 2015. The list of studies was updated with a second round of search in March 2016.

### Inclusion and exclusion criteria

An article had to fulfill the following criteria to be eligible for inclusion: (1) the subjects of study had to be healthy children or adolescents below the age of 18; (2) the studies had to utilize quantitative methods (surveys and experiments were both included), with statistical significance being reported; (3) the independent variable(s) must contain one parenting practice or style; (4) the dependent variable(s) were either food cognitions such as attitudes (implicit and explicit), liking, preference or social norms, or food consumption choice and/or intake of fruits and vegetables or SSBs.

Articles that were excluded were: (1) articles not published in English; (2) studies that involved only overweight or obese parents and/or children; (3) studies that stratified analyses according to BMI without providing overall effect sizes; (4) studies that examined the dependent variable(s) of eating problems and styles such as eating disorders, disordered eating, and emotional eating, as well as BMI; and (5) intervention studies.

### Analytical approach

In the first phase, a three-step process was utilized to identify relevant studies for the systematic review. First, the retrieved papers were screened and shortlisted using their titles. Next, the abstracts of the shortlisted articles were screened in another round of shortlisting. Two reviewers were involved in shortlisting the articles. Overall, percent agreement between the reviewers were at 92%. All disagreements were discussed between the reviewers and resolved by revisiting the inclusion/exclusion criteria and by coming to a consensus. Finally, entire articles were retrieved from the database and screened, with eligible studies being retained for the review. The associations between parenting factors and child eating outcomes were represented with “+” for a significant positive association, “-” for a significant inverse association, and “0” for a null association. Associations with a reported *p* value of < 0.05 were considered significant. In studies with univariate and multivariate results, the associations considered were taken from the multivariate analysis. Studies with statistical analyses indicating the significance level were included. Mediation and moderation effects are stated in the final column of Additional file 1: Table S1. In stratifying the analysis according to child age, Piaget’s three stages of cognitive development in children was utilized [22]. Parental factors that saw less than 2 studies conducted within a developmental stage were omitted from the analysis.

As there were no standardized terminologies for parental factors, with researchers often utilizing conflicting definitions with regard to similar terms, we attempted to develop a list of overarching parental constructs by examining each study’s operationalized measurement items. First, we examined all the measures used across every study. Next, we grouped the measurement items into arbitrarily named parental practice variables based on face validity. When operationalized measurement items grouped together came from a wide range of differently named constructs (across different researchers), the variable they represent were given a new term in order to avoid confusion (e.g., *parental encouragement through rationale* and *parent nutrition teaching* were classified under *active guidance*). On the other hand, when a group of measures representing a variable were derived from a commonly used term, the term was retained (e.g., *modeling).* Once all the factors were identified and named, two reviewers classified the measures in each included study based on the identified constructs. Percent agreement between the two reviewers were at 91% overall. Disagreements were resolved through a discussion between the reviewers.

**Fig. 1:**
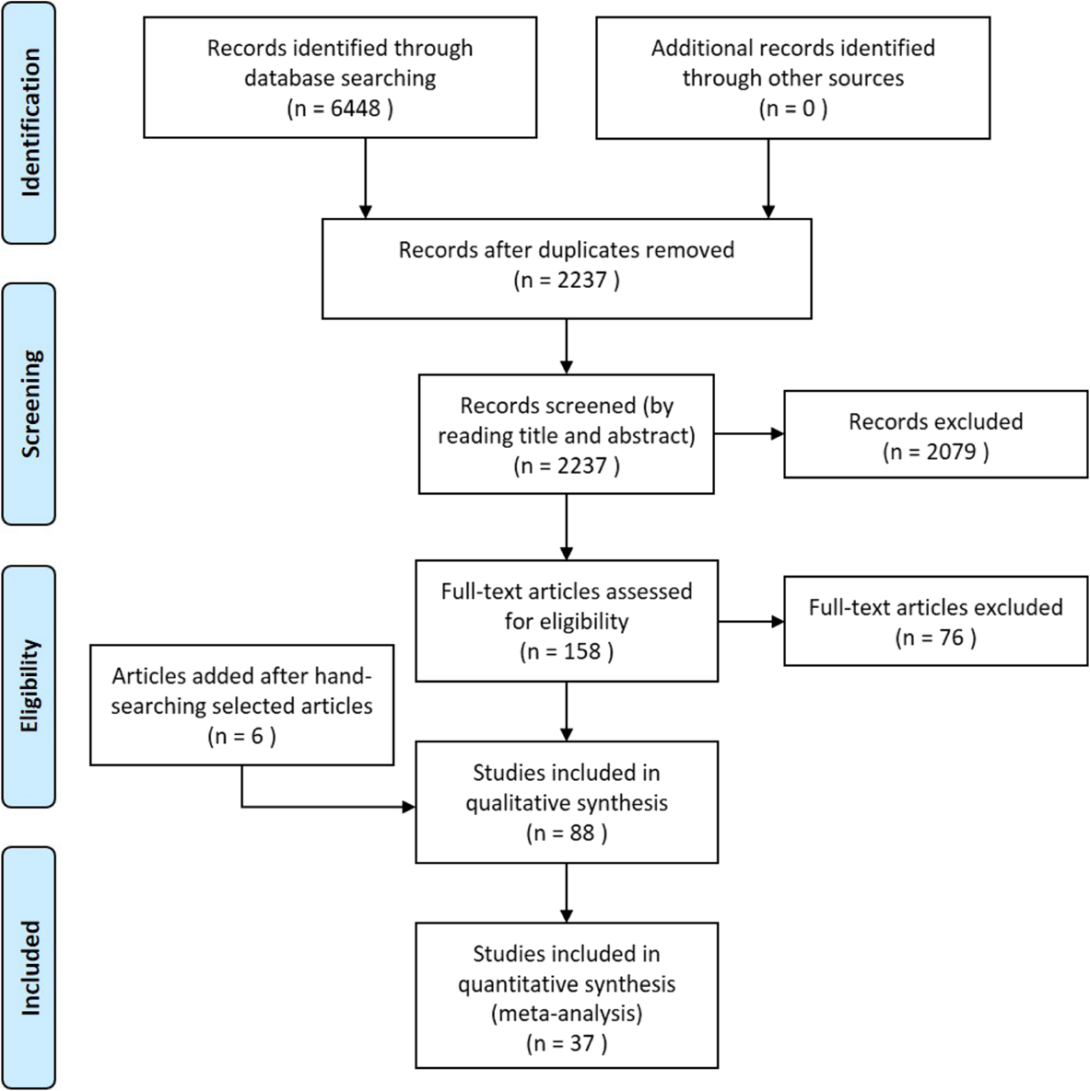
PRISMA flow diagram

In the second phase, a meta-analysis was conducted for studies that were eligible for the calculation of effect sizes using the Comprehensive Meta-Analysis 2 software. In order for studies to be included in the meta-analysis, the quantitative information required for computation of overall effect size, such as sample sizes, means, correlations, odds ratios and test statistics must have been available. Studies without these required information were excluded. In order to generate overall effect sizes, data from studies that included correlations, means with standard deviations, or odds ratios were converted into Pearson’s product–moment correlation scores, resulting in commensurable effect sizes across studies. The conversions were executed using established formulas [23]. Studies utilizing Spearman correlation coefficients were also included, as Spearman correlation coefficients are equivalent or only slightly smaller as compared to Pearson’s product–moment correlation scores [24]. These correlation coefficients were then weighted according to the corresponding study’s sample size and standard error, resulting in a weighted correlation score [25]. Following that, the overall effect sizes of each predictor and outcome were calculated by averaging the weighted correlation coefficients across the eligible studies. As some studies utilized a combination of food items within a single category as dependent variables (e.g., fruits and vegetables as separate measures), the meta-analysis was conducted by averaging the correlations between an independent variable and the dependent variables within a category, so that only a single correlation score is computed for either healthy or unhealthy food consumption.^1^ This meta-analysis was conducted using random effects models. An *a priori* assumption guided this choice. Since studies included in this analysis were not identical, it was not possible to assume a true effect size across the studies. The studies were conducted independently across different age groups of children, and across different cultural contexts, indicating that the use of random effects models was more appropriate in the analysis [26]. Figure 1 displays a PRISMA flowchart illustrating the systematic process of conducting the review.

## Results

A total of 6448 titles were located from the literature search (Web of Science = 1692; ERIC = 190; PsycINFO = 708; PubMED = 3858). After removing duplicates, there were a total of 2237 unique titles potentially relevant for inclusion in this review. After screening the titles, 556 articles were retained for abstract screening. Following that, 158 full-text articles were retrieved and scanned in their entirety for eligibility, with 82 articles being retained for analysis. Six articles were added after hand searching the reference lists of relevant articles, resulting in a total of 88 articles utilized in this review. A summary of the studies can be viewed in Additional file 1: Table S1.

### Characteristics of included studies

Most of the studies were cross-sectional studies (*n* = 66), with the rest being longitudinal (*n* = 14), experimental (*n* = 5), and quasi-experimental (*n* = 3). 49% of the studies involved children in the pre-operational stage (ages 2 to 6; *n* = 43), 33% involved those in the concrete operational stage (ages 7 to 11; *n* = 29), while only 16% of the studies involved those in the formal operational stage (ages 12 to 18; *n* = 16)^2^ [22]. Most studies were situated in the US, Europe, and Australia, with the exception of three studies: one being situated in Israel, one in Costa Rica, and another in Hong Kong. The spread of parent-report (*n* = 33), child-report (*n* = 28), and parent-child matched samples (*n* = 27) were evenly matched. Despite this, most studies with parent-report measures consisted of a large number of female participants.

With regard to specific target food items being examined, 64% of studies examined parental influence on fruits/vegetables consumption (*n* = 56), 24% examined SSBs consumption^3^ (*n* = 21), 51% examined some form of unhealthy eating^4^ (*n* = 45), and 10% examined healthy foods consumption^5^ (*n* = 9). Since specific food items (eg. fruits/vegetables, SSBs) were measured in the composite measures of general healthy and unhealthy food consumption, the subsequent analysis of the effects of parenting on child food consumption considered them under the concepts of healthy (promotive) and unhealthy (preventive) food consumption.

A total of 10 parental factors were identified from the literature. They included context-specific parental practices of restrictive guidance (or rule-making) (*n* = 48), modeling (or parents’ own food consumption behavior) (*n* = 37), availability (or the control of availability) (*n* = 36), parental pressure to eat (*n* = 25), food as reward (*n* = 12), rewarding food consumption (*n* = 10), rewarding food consumption with praise (*n* = 9), accessibility (*n* = 6), and active guidance (or verbal education and encouragement) (*n* = 5). Meanwhile, the effects of general parenting styles were examined in 18% of the studies (*n* = 16).

### Context-specific parental practices and child food consumption

#### Restrictive guidance

Restrictive guidance is defined as the frequency with which parents set limits, rules, or restrictions regarding food consumption. Closely related to the parental mediation dimension of restrictive mediation [27], this includes a range of overt parental restriction constructs such as rule-making and parental overt control. Among the included studies, 33 examined the relationship between parental restrictive guidance and healthy eating such as fruits and vegetables intake. Amongst these, 13 indicated a positive relationship with healthy eating, three yielded a negative relationship, whilst 17 indicated a non-significant relationship. For unhealthy eating such as the consumption of SSBs, restrictive guidance was found to reduce consumption in 16 out of 38 studies. Eight studies suggested that parental restrictive guidance is associated with *higher* consumption of unhealthy foods among children, while 14 studies indicated a null relationship. This suggests a large amount of heterogeneity in the relationship between restrictive guidance and both healthy and unhealthy child food consumption behaviors.

#### Modeling

Modeling was measured in two ways across the literature. First, modeling was measured as parental intake of a target food item. In such a case, a positive relationship between parent and child intake was indicative of a significant modeling effect. Second, modeling was measured as the frequency in which parents eat healthily and demonstrate the benefits and pleasure of eating healthily in front of children. In both cases, modeling was consistently found to be associated with child eating. Among the 31 studies that examined modeling and healthy food consumption, 28 found a significant positive relationship, while three saw no significant association. Among the 16 studies that examined modeling and unhealthy food consumption, 13 showed a significant positive association, while three saw no significant association. This suggests that the effects of parental modeling on child food consumption behavior are homogenous and significant.

#### Parental control of availability

Parental control of availability simply refers to whether a particular food is available at home. Since children develop food preferences through consistent exposure to foods [28–30], the availability of food would be crucial in determining whether children develop healthy food preferences rather than energy-dense food preferences such as SSBs. Availability was measured in two ways across the reviewed studies. First, it was measured with regards to actual availability of a target food item (this can be measured as availability or non-availability of a target food). Second, it was measured as a parental strategy that involves parents controlling the availability of unhealthy foods in a covert manner [31–33].

Among the 27 studies examining availability of healthy foods and healthy foods consumption, an overwhelming 19 studies indicated a positive association, whilst 5 indicated a null relationship. Likewise, availability of healthy food, non-availability of unhealthy foods, as well as parental control of availability of unhealthy foods, were associated with decreased unhealthy eating in 18 out of 22 studies. Three studies indicated a null relationship. These results suggest that availability or control of food availability can be a consistent predictor of child food consumption.

#### Pressure to eat

Parents can also pressure children into eating more food [34]. This practice refers to parents utilizing verbal communication to try and persuade their children to consume more food. Pressure can manifest in a parent asking his or her child to clean up their plates, even if the child says he or she is not hungry [35]. Underlying parental pressure to eat is a fear that a child is not consuming enough food. Although parents’ intention when utilizing pressure is to encourage sufficient nutrient intake, some researchers have argued that it can have the opposite effect, leading to lower fruits and vegetables intake [36, 37]. Of the 22 studies that examined pressure to eat and healthy food consumption, 14 showed no significant associations. Six studies found significant negative associations, where pressure was shown to be associated with less healthy food consumption. Only two studies found a positive association. With regards to unhealthy food consumption, eight studies showed that pressuring a child to eat was associated with significantly higher unhealthy food intake, while 13 studies showed that there were no significant associations.

#### Food as reward

Parents might also utilize food as a reward when children exhibit desirable behavior. Three studies found a negative association between using food as reward and healthy food consumption, with the large majority of studies (*n* = 7) finding no significant relationships between the two variables. In contrast, six out of ten studies that examined the influence of food as reward on unhealthy eating found a positive relationship. As unhealthy foods such as sweet snacks are most often used as the reward item, this practice tends to increase preferences for unhealthy foods [38, 39]. However, less is known about how the utilization of healthy foods such as fruits and vegetables as reward can influence child food consumption outcomes.

#### Rewarding food consumption materially

Rather than using food as a form of reward, parents can sometimes reward healthy food consumption with material or playtime rewards [39, 40]. According to self-determination theory [41, 42], providing extrinsic rewards for an activity can potentially decrease the motivation and desire to perform the activity. When a reward is provided for eating a certain food, a child will tend to devalue the instrumental activity performed to receive the reward, as the child will see the activity as a means to an end [43]. In other words, if eating healthy food is the only way a child can get to play outside, the child might see it as a chore, and subsequently hold negative mental associations, feelings, and cognitions about the food.

Out of 10 studies, most studies (*n* = 6) found no significant relationships between rewarding food consumption and child healthy eating. Three studies found a positive association, while one study found a negative association. Among the four studies that examined the relationship between rewarding food consumption and unhealthy eating, one study found a positive association. This positive association could be due to a single item in the composite measure of rewarding food consumption belonging conceptually closer to using food as reward (e.g., dessert as contingent upon eating something the child doesn’t like) [44].

#### Rewarding with praise

Parents can reward children with praise rather than material or hedonic rewards. Some researchers have suggested that praise is qualitatively different from other types of rewards [40], likely because praise fulfills and fosters the intrinsic needs of relatedness, competence, and autonomy, as compared to extrinsic rewards. Among seven studies that examined the relationship of praise with healthy food consumption, four found positive relationships, while three yielded non-significant results. With regards to unhealthy eating, one study found that praise was associated with savory snack consumption among girls only, while the other studies (*n* = 4) found no significant relationships.

#### Accessibility

Parental control of food accessibility refers to “*whether the foods are prepared, presented, and/or maintained in a form that enables or encourages children to eat them* (p. 26) [45]”. Six studies examined the relationship between making food accessible and child fruits/vegetables intake. Four studies indicated a positive relationship, while two indicated a null relationship, suggesting that accessibility can potentially be a useful parental strategy in encouraging healthy eating.

#### Active guidance

Active guidance or education is defined as the degree which parents actively discuss, verbally interact, and instruct their child with regards to food. Only five studies examined the influence of active guidance on child food consumption. In these studies, the measures indicating active guidance were encouragement through rationale (e.g., eating vegetables is good for you) [44], or nutritional teaching regarding types of food [46]. Amongst the studies, one found a positive relationship between active guidance and vegetables consumption, as well as a negative relationship between active guidance and SSBs consumption. However, the four other studies have indicated a non-significant relationship between active guidance and healthy or unhealthy eating.

### General parenting styles and moderation effects

Parenting researchers recognize that individual context-specific parenting behaviors are a part of a complex milieu of other parenting behaviors, so no individual parenting practice (eg. such as the use of active guidance) can be isolated and tested for its influence without considering other facets of parenting [15]. In view of this, general styles of parenting can be viewed as a circumstantial construct that would moderate the influence of context-specific parenting behaviors such as active parental guidance, using food as reward, pressuring children to eat and so on [47–49]. This perspective adopts the view that context-specific parenting practices can have different effects when employed by families who utilize a different parenting style [50].

In general, most studies reviewed examined the direct relationship between parenting variables and child food consumption outcomes. Only approximately 10% (*n* = 9) of all the articles reviewed examined the *moderating* influence of general parenting styles, indicating a lack of empirical understanding about the complex influence of parenting styles on the effects of parenting practices. Amongst those that examined parenting styles as a moderator, one noted that restrictive guidance situated in an authoritarian parenting style showed stronger effects on fruits intake, whilst availability situated in an authoritative parenting style showed stronger effects on fruits and vegetables intake [51]. Another study utilizing parental *feeding* style as a moderator supported this, as they found that restrictive guidance led to lower intake of low-nutrient-density foods among less permissive parents [52]. One study found that restrictive guidance had stronger positive associations with healthy eating, and stronger negative associations with unhealthy eating when parental warmth is high, suggesting that whilst demandingness/control might lead to more effective restrictive guidance, warmth can also play a role in moderating the effects in the same direction [53]. Another echoed these findings, as they found that restrictive guidance and availability were more strongly associated with lower child SSBs intake amongst children with highly responsive, as well as moderately demanding parents [54]. Other studies contradicted these findings. Specifically, one study found that children under an indulgent parental feeding style (low in demandingness, high in responsiveness) saw greater positive relationship between restrictive guidance and fruits and vegetables consumption [49]. Likewise, two other studies found that restrictive guidance was associated with lower SSBs intake only when behavioral control was low among parents [55, 56].

Other than its moderating effects on restrictive guidance, one study found that the strongest associations between modeling and child fruit consumption occurred among children who were under a highly controlling parenting style [55]. Another study also found that controlling the availability of food was also more effective in reducing SSBs intake amongst children of parents who reported higher control [56]. Of all nine studies, only one study found no moderating effect from parenting styles [57].

### Cognitive mediators of food consumption

Five studies sought to examine the influence of parenting on food cognitions such as preference [58], liking [59, 60], subjective norms [61], desire [62], and attitudes [57, 61]. Although all five studies indicated some level of significant relationships between parenting variables and food cognitions, mediation through these cognitions were not tested statistically. Only one reviewed study examined potential psycho-social *mediators* between parenting variables and child food consumption [63]*.* In the study, the researchers found that self-efficacy partially mediated the effects of modeling on fruits and vegetables consumption.

### Differences by child age

Eighty-six studies were included in the stratified analysis of studies based on child age. Two studies were omitted as there was insufficient information regarding the mean age of the children being referred to available within the full-text article. Table 1 summarizes the findings of the studies being reviewed, according to child age. Interestingly, some age differences can be observed.

**Table 1:** Systematic review of parental predictors on child healthy and unhealthy food consumption by age

	Healthy Food			Unhealthy Food		
Predictors	Pos.	Neg.	N.S.	Pos.	Neg.	N.S.
	Pre-operational stage (Age 2 to 6)					
Restrictive Guidance	35% (6)	12% (2)	53% (9)	33% (7)	29% (6)	38% (8)
Active Guidance	0%	0%	100% (2)	-	-	-
Availability	78% (7)	0%	22% (2)	100% (7)	0%	0%
Accessibility	67% (2)	0%	33% (1)	-	-	-
Modeling	100% (14)	0%	0%	67% (2)	0%	33% (1)
Pressure to eat	14% (2)	36% (5)	50% (7)	55% (6)	0%	45% (5)
Rewarding food consumption	50% (3)	0%	50% (3)	-	-	-
Reward with verbal praise	80% (4)	0%	20% (1)	0%	0%	100% (2)
Food as reward	0%	25% (2)	75% (6)	71% (5)	0%	29% (2)
	Concrete operational stage (Age 7 to 11)					
Restrictive Guidance	40% (4)	10% (1)	50% (5)	9% (1)	45% (5)	45% (5)
Active Guidance	50% (1)	0%	50% (1)	0%	50% (1)	50% (1)
Availability	92% (12)	0%	8% (1)	88% (7)	0%	13% (1)
Accessibility	67% (2)	0%	33% (1)	-	-	-
Modeling	87% (13)	0%	13% (2)	78% (7)	0%	22% (2)
Pressure to eat	0%	14% (1)	86% (6)	14% (1)	0%	86% (6)
Rewarding food consumption	0%	0%	100% (3)	0%	0%	100% (3)
Reward with verbal praise	0%	0%	100% (2)	0%	0%	100% (2)
Food as reward	0%	50% (1)	50% (1)	50% (1)	0%	50% (1)
	Formal operational stage (Age 12 to 18)					
Restrictive Guidance	0%	0%	100% (3)	0%	83% (5)	17% (1)
Active Guidance	-	-	-	-	-	-
Availability	67% (2)	0%	33% (1)	60% (3)	20% (1)	20% (1)
Accessibility	-	-	-	-	-	-
Modeling	50% (1)	0%	50% (1)	100% (4)	0%	0%
Pressure to eat	-	-	-	-	-	-
Rewarding food consumption	-	-	-	-	-	-
Reward with verbal praise	-	-	-	-	-	-
Food as reward	-	-	-	-	-	-

Values in brackets reflect the number of studies that found either a positive, negative, or non-significant effect

First, all three studies that examined restrictive guidance among older children in the formal operational stage (FOS) found no significant associations with healthy food consumption. This was in contrast to studies with younger children in the pre-operational stage (POS) and concrete operational stage (COS), where a large amount of heterogeneity exists, and with a substantial proportion of studies finding a significant positive relationship (40 and 35% respectively). Interestingly, a greater proportion of studies showed that restrictive guidance had a negative relationship with unhealthy food consumption among children in FOS (83%), as compared to those in the COS (45%) and POS (29%).

Unsurprisingly, a greater proportion of studies among older children (COS compared to POS) found a positive relationship between active guidance and healthy food consumption, and a negative relationship between active guidance and unhealthy food consumption. This suggests that active guidance might be more useful in shaping food consumption behaviors with children that are at a more advanced developmental stage. Unfortunately, there were no studies that involved children in the FOS for comparisons.

More studies appear to show the undesirable effects of pressuring a child to eat among younger children. 36% of the studies involving children in POS found a negative relationship with healthy food consumption, while 55% found a positive relationship with unhealthy food consumption. However, for samples involving older age groups, a vast majority of studies found no significant relationship between pressuring and both types of food consumption. Rewarding food consumption either with praise or with tangible rewards appear to be more effective in promoting healthy eating among younger children in the POS, with 50% of the studies finding a positive relationship between rewarding food consumption and healthy food consumption, and 80% finding a positive relationship between rewarding with praise and healthy food consumption. However, these significant findings were non-existent among those in the COS.

### Meta-analysis findings

In addition to the preceding systematic review, a smaller subset of 37 studies with available statistics was utilized to conduct a quantitative meta-analysis. Except for the relationship between accessibility and unhealthy food consumption, all relationships had at least two eligible studies, allowing us to compute a meta-analytic effect size for each relationship between variables. As illustrated in Table 2, five out of the nine parental communication variables had a statistically significant weighted correlation with child *healthy* food consumption. Specifically, active guidance (*r* = .15, *p* < .001), availability (*r* = .24, *p* < .001), modeling (*r* = .32, *p* < .001), and verbal praise (*r* = .15, *p* < .05) were significantly and positively correlated with child healthy food consumption. The effect sizes were small to medium according to guidelines developed by Cohen [64, 65]. In addition, pressuring children to eat had a small but significant negative correlation with child healthy food consumption (*r* = −.04, *p* < .05). Restrictive guidance, accessibility, and rewarding food consumption were not significantly correlated with child healthy food consumption.

**Table 2:** Meta-analysis of parental predictors of child healthy food consumption (32 unique studies included)

Predictors	K	N	Effect Size	95% CI		Q
				Low	High	
Restrictive Guidance	13	9628	.05	−.01	.11	70.984***
Active Guidance	2	1142	.15***	.09	.21	0
Availability	15	23825	.24***	.17	.31	298.711***
Accessibility	3	10704	.10	−.04	.24	48.842***
Modeling	18	20104	.32***	.25	.39	291.395***
Pressure to eat	11	10808	−.04*	−.08	.00	27.025**
Rewarding food consumption	4	4124	.03	−.02	.07	6.265
Reward with verbal praise	4	3381	.15*	.00	.30	54.611***
Food as reward	3	1509	−.07*	−.13	−.01	2.245

Effect size calculations were based on random effects model

*K* number of studies, *N* total sample size for all studies, *Effect Size* Pearson’s r, *CI* confidence interval, *Q* heterogeneity in effect sizes between studies

*p < .05, **p < .01, *** *p* < .001

With regards to child *unhealthy* food consumption, six out of the nine identified parental communication variables were significantly correlated with unhealthy food consumption (See Table 3). Specifically, restrictive guidance (*r* = −.11, *p* < .01), and verbal praise (*r* = −.04, *p* < .05) had small but significant negative correlations with child unhealthy food consumption. On the other hand, availability (*r* = .34, *p* < .001), modelling (*r* = .35, *p* < .001), pressure to eat (*r* = .04, *p* < .05), and using food as reward (*r* = .14, *p* < .05) were positively correlated with child unhealthy food consumption. Active guidance and rewarding food consumption were not significantly correlated with child unhealthy food consumption.

**Table 3:** Meta-analysis of parental predictors of child unhealthy food consumption (23 unique studies included)

Predictors	K	N	Effect Size	95% CI		Q
				Low	High	
Restrictive Guidance	15	14539	−.11**	−.17	−.04	193.800***
Active Guidance	2	1142	−.04	−.24	.17	10.664***
Availability	6	3421	.34***	.21	.46	56.146***
Accessibility	–	–	–	–	–	–
Modeling	5	1593	.35***	.22	.47	28.800***
Pressure to eat	9	8686	.04*	.00	.08	17.856*
Rewarding food consumption	3	2569	.06	−.04	.15	8.939**
Reward with verbal praise	4	3381	−.04*	−.07	−.01	2.744
Food as reward	4	1644	.14*	.03	.25	7.137

Effect size calculations were based on random effects model

*K* number of studies*, N* total sample size for all studies*, Effect Size* Pearson’s r*, CI* confidence interval*, Q* heterogeneity in effect sizes between studies

*p < .05, **p < .01, *** p < = .001

### Meta-analysis moderated by child age

In addition to the systematic review suggesting some age differences, a significant *Q* statistic suggests that there was heterogeneity in the effect sizes between studies [66]. As such, examining age as a moderator in the analysis could reveal potential differences. Among the nine parental communication variables, active guidance, rewarding food consumption and using food as reward did not have heterogeneous results across study in the context of child *healthy* food consumption, thus they were excluded from the analysis. Likewise, reward with verbal praise and food as reward were excluded from the analysis in the context of child *unhealthy* food consumption due to it fulfilling the assumption of homogeneity. Additionally, active guidance was excluded from the analysis as there were only two studies available for moderator analyses. Table 4 and 5 summarizes the results of the meta-analysis moderated by child age.

**Table 4:** Moderator analysis of child healthy food consumption

Predictors	Moderator		K	Effect size
Restrictive Guidance	Age Group	POS	8	.03
		COS	3	.06
		FOS	2	.08
Availability	Age Group	POS	6	.16**
		COS	8	.26***
		FOS	1	.53***
Accessibility	Age Group	POS	1	.26***
		COS	2	.04
Modeling	Age Group	POS	9	.34***
		COS	7	.24***
		FOS	1	.42***
Pressure	Age Group	POS	8	−.06*
		COS	3	−.01
Reward with Verbal Praise	Age Group	POS	2	.26***
		COS	2	.04

Effect size calculations were based on the random effects model

*K* number of studies, *Effect Size* Pearson’s r. *p < .05, **p < .01, ***p < .001

**Table 5:** Moderator analysis of child unhealthy food consumption

Predictors	Moderator		K	Effect size
Restrictive Guidance	Age Group	POS	9	−.03
		COS	3	−.19***
		FOS	3	−.20*
Availability	Age Group	POS	5	.37***
		COS	1	.22***
Modeling	Age Group	POS	2	.48***
		COS	3	.27***
Pressure	Age Group	POS	6	.05
		COS	3	.04
Reward with Verbal Praise	Age Group	POS	2	−.08**
		COS	2	.−.02

Effect size calculations were based on the random effects model

*K* number of studies*, Effect Size* Pearson’s r. *p < .05, **p < .01, ***p < .001

The results showed that although availability had a significant positive relationship with healthy food consumption across all age groups, it had a stronger association with healthy food consumption among older children. However, this ought to be read with caution, as among the FOS group, only one study was utilized in the analysis. Echoing the results of the systematic review analyses stratified by child age, the undesirable effects of parental pressuring on healthy food consumption was found only among younger children, and not older children. Likewise, the positive associations between rewarding with verbal praise and healthy food consumption existed only among younger children, and not older children.

With regard to unhealthy food consumption, the results indicated that restrictive guidance was more effective in reducing unhealthy food consumption among older children. In addition, rewarding desirable eating behavior with verbal praise had a negative association with unhealthy food consumption only among younger and not older children.

## Discussion

The systematic review of parental factors and child promotive and preventive food consumption outcomes yielded a total of 10 potential parental factors, 9 of which captured context-specific parental factors, and one of which referred to parenting styles as a moderating factor. Of these, availability or the control of availability of food items, along with parental modeling were consistently associated with both desirable and undesirable food consumption cognitions and consumption. This was supported by both the systematic review and the meta-analysis. First, meta-analytic procedures found that they had the strongest correlations with both child healthy and unhealthy food consumption. Second, the systematic review found that a vast majority of studies support the direction of relationship as indicated in the meta-analysis. Parents availing food can increase consumption from the possibility that children eat whatever that is available to them.

In addition, parental modeling could possibly convey attitudinal, norms-based, and self-efficacy beliefs to children, which drives consumption behavior [67]. According to social cognitive theory, modeling refers to the process whereby an individual (in this case a child) learns through his or her observations of another person performing a behavior [68, 69]. Specifically, it is hypothesized that a child will imitate or adopt behaviors when they observe an influential role model in their lives (e.g., a parent) perform said behaviors. Modeling effects take place because observations of other people eating will influence their own beliefs about what to eat, and how much is appropriate to eat [70]. In addition, some researchers have defined modeling as a type of parental communication strategy, consisting of parental encouragement for their children to adopt their own behaviors intentionally through overt verbal communication and covert non-verbal strategies [71–73]. Specifically, modeling can also occur when parents explicitly display to their children that they enjoy certain food items, leading to a child’s vicarious learning about the enjoyment of eating certain food types. Such explicit displays of eating behaviors are used as a parenting strategy to promote attitudes toward, and subsequently, consumption of certain foods among children. Our systematic review reveals that both types of modeling are similarly associated with more desirable eating behaviors.

Restrictive guidance was found to be negatively associated with unhealthy food consumption, while its association with healthy food consumption was mixed. This was supported by both the systematic review and meta-analysis. When the analyses were stratified by age, an interesting pattern emerged. While the association of restrictive guidancewith healthy food consumption was mixed for younger children, studies with children older than 12 found no evidence for such an effect. This suggests that restrictive guidance might be ineffective in promoting desirable food choices among older children. However, it is interesting to note that the relationship between restrictive guidance and unhealthy food consumption was more powerful among older children, as compared to those in the POS. It is possible that younger children in the POS are less able to follow rules or limits set out by parents due to limited self-regulation capabilities [74]. Future research is suggested to better understand why this phenomenon exist.

Next, using food as reward was found to be related to higher levels of unhealthy eating among children. This was supported in both the systematic review and meta-analysis. This is likely due to the fact that foods used as rewards are often unhealthy. By rewarding children’s behavior with these unhealthy food items, children might associate these unhealthy items as positive and valuable, hence consuming more of them when they have the chance [75].

Pressuring children to eat can lead to effects unintended by the parents. Although a small number of studies found a positive association between pressuring and healthy eating, a larger number of studies found a negative association. In addition, seven studies showed that pressuring can be associated with higher unhealthy food consumption. This is also played out in the meta-analysis, with pressuring being shown to have a small negative correlation with healthy food consumption and small positive correlation with unhealthy food consumption. However, age-stratified analyses revealed that these unintended effects tend to be limited to younger children, as the effects are non-significant in studies involving older children.

The evidence that rewarding food consumption can influence child food consumption was weak, as most studies included in the systematic review showed no significant effects. The meta-analysis also showed no significant correlation between rewarding food consumption and child food consumption. On the other hand, a larger proportion of studies found that verbal praise might lead to higher healthy foods consumption, indicating that rewarding with material rewards and praise are distinct practices with different outcomes. However, it is important to note that rewarding with praise appeared to be more useful with younger children in the POS, with the effects diminished in studies involving older children.

Although the number of studies examining accessibility was small, the majority of studies in the systematic review indicated that accessibility can have a positive influence on healthy foods consumption. This was not supported in the meta-analysis, as there was a non-significant correlation between accessibility and child healthy food consumption. One reason for this discrepancy might be due to the limited number of studies included in the meta-analysis.

Interestingly, active guidance received very little attention from researchers, with only 6% of the studies treating it as a potential predictor of child food consumption. Despite this, research in the domain of parental mediation has found active discussion to be an effective parental socialization technique in influencing desirable child outcomes [76]. The meta-analysis results suggest that these effects might translate well into the child food consumption context, as there was a small to medium, positive, and significant relationship between active guidance and child *health* food consumption. One reason for the modest findings could be that the existing measures are inadequate in capturing the concept of active guidance in its entirety. Existing concepts that were used as proxies for active guidance in this study were *teaching about nutrition* and *encouragement through rationale* [44, 77]. These constructs are more closely related to *factual* parental guidance, as opposed to *evaluative* parental guidance. *Factual* active guidance refers to informing children with technical knowledge. Such techniques have been found to be ineffective in mitigating negative media effects on children [78, 79]. *Evaluative* active mediation, on the other hand, reflects parents’ attempts to provide either positive or negative opinions about a topic, and are considered to be a more effective strategy [79]. Examining the influence of evaluative, rather than factual parental guidance, might yield stronger associations. A second reason could be that there were insufficient studies examining the effect of active guidance on older children. Our age-stratified systematic review analysis found that the beneficial effects of active guidance were more pronounced in studies involving older children in COS, as compared to those in POS. However, only 3 studies out of the 88 unique studies examined active guidance among children older than 7.

### Opportunities for future research

The systematic review has identified several gaps in the study of parental communication and child food consumption. First, although some parental communication practices were consistently linked with child food consumption outcomes (e.g., modeling and availability), many of the other parental communication practices show mixed results (e.g., restrictive guidance) and some are severely understudied (e.g., active guidance). More research needs to be conducted in these areas to ascertain the relationships between parental communication and child food consumption behavior. Second, the concept of active guidance is currently underdeveloped and understudied. Although active discussions have been shown to be an effective parental communication strategy in socializing children to more desirable outcomes in other contexts [76], there remains a lack of understanding of how parents’ active verbal discussions with children apply in the food consumption context. Specifically, there has been a lack of conceptual explication of parental guidance in the food consumption context, with existing studies not taking into account the different facets of parental guidance. Existing studies have examined active parental guidance from the perspective of factual guidance (e.g., teaching about nutritional facts). As previous studies have found factual guidance to be less effective than evaluative guidance, future studies should attempt to examine evaluative guidance as a correlate. Existing studies also do not take into account how positive or negative active guidance can influence outcomes. As healthy eating choices are to be promoted, and unhealthy eating choices are to be discouraged, active guidance as dichotomized into positive vs. negative can have differential impact. Positive active guidance might be more effective in promoting healthy eating, and less effective in discouraging unhealthy eating. This is suggested in our meta-analysis results where active guidance (which reflect a more positive construct in the operationalization across studies) was found to be correlated with healthy eating but not unhealthy eating. Third, theoretically-driven studies of parental communication on child food consumption, which takes into account psychosocial mediating variables, are severely lacking. The systematic review found that only five studies attempted to understand child food consumption from such a perspective. Without considering the social psychological processes of the child, it is difficult to ascertain the pathways of how certain parental practices are influencing child food consumption behavior. This means that current understanding of parental communication on child food consumption is incomplete.

### Limitations

There are several limitations of this study that should be taken into account when drawing conclusions from the results. First, the review was conducted using a non-exhaustive list of studies. Although effort was made to be comprehensive in the retrieval of articles by searching through four major databases and hand-searching relevant articles, there could have been some studies that were missed out on. To minimize this, we hand-searched the reference list of every selected study, which helped ensure greater comprehensiveness in the systematic review. Despite this, it is important to note that the conclusions are drawn from overall trends across studies, rather than from singular studies, rendering a small number of missed articles less consequential. Second, most of the studies were limited to populations in the west. As nuances in parent-child relationships might exist across cultures, the findings might not generalize across to cultures outside of the west. Lastly, there is a lack of uniformity in measuring a number of these parental variables. Despite this, our efforts to synthesize and categorize the variables have yielded strong agreement rate between two coders. Nonetheless, it is important to note that in several of these studies, there is strong heterogeneity in terms of operationalizing some of these variables, limiting their comparability.

## Conclusion

There is a wide variety of behaviors that parents engage in to either promote or prevent certain child food consumption behaviors. Some behaviors, such as parents’ own food consumption behavior, and availing certain types of food, have been shown to be strong correlates of child food consumption behavior. On the other hand, some behaviors such as active and restrictive guidance, are effective only in certain contexts; active being more effective in encouraging fruits and vegetables consumption, while restrictive guidance is more effective in discouraging unhealthy eating such as SSBs consumption. Despite this, there appears to be a number of research gaps that needs to be filled. Researchers in the field can take note of the opportunities for future research highlighted from this comprehensive study. In doing so, we can better understand what constitutes an ideal family environment that helps improve the wellbeing of our children.

## Additional files

Additional file 1: Table S1. Summary of studies included in the systematic review. (PDF 402 kb)

Additional file 2: Meta-analysis dataset. Cleaned meta-analysis dataset. (XLSX 15 kb)

Additional file 3: Database of all studies included in this review. This database allows for filtering of the studies according to the age group of children. (XLSX 47 kb)

## Footnotes

1 For example, if Study A examined the effects of restrictive guidance on fruits *and* vegetables consumption and found their correlations as *r* = .15 and *r* = .25, the correlations would be averaged ($\frac{.15+.25}{2}$ = .20), such that a single *r* = .20 is used to represent restrictive guidance of food consumption’s correlation with *healthy* food consumption.

2 Articles were grouped into these three groups of developmental stages using the mean age of the children under study

3 Including high-energy drinks

4 Including unhealthy snacks, low-nutrient density food, junk foods, and non-core foods; some of these composite measures of unhealthy eating consisted of a component of SSBs consumption

5 Including core-foods and healthy snacking (eg. fruits as snacks)
