# Supplementary material for: The influence of parental practices on child promotive and preventive food consumption behaviors: a systematic review and meta-analysis
Source: Int J Behav Nutr Phys Act. 2017 Apr 11;14:47. doi: 10.1186/s12966-017-0501-3 (PMC5387370; doi:10.1186/s12966-017-0501-3)
Supplement: Supplementary file 1 — Summary of studies included in the systematic review. (PDF 402 kb) [file 12966_2017_501_MOESM1_ESM.pdf]

Study	Author(s)	Year	Outlet	Design	N	Participants	Independent Variable(s)	Dependent Variable (s)	Evidence of Effect	Moderators/ Mediators/ Comments
1	Alsharairi & Somerset	2015	Ecology of Food and Nutrition	Longitudinal survey	4,310	Australian children aged 4 to 9	Authoritarian parenting style	F&V intake	-	
							Maternal demandingness	F&V intake	-	
							Paternal Responsiveness	F&V intake	+	
							Permissive father	F&V intake	+ (later)	
							Authoritative fathers	F&V intake	+ (later)	
							Authoritative mothers	F&V intake	+ (later; girls)	
2	Amuta et al	2015	Food and Exercise in Rural Areas	Cross-sectional survey	298	Predominantly mothers living in rural areas in the US, elementary school children, child age not available	Restrictive guidance (during mealtime)	F&V intake	0	
3	Arredondo et al	2006	Health Education Research	Cross-sectional survey	812	Predominantly Latino mothers in the US, with child mean age of 6 (*SD* = 0.94)	Reward (with praise)	Healthy eating	+	Gender moderated some of the effects
								Unhealthy eating	-	
							Pressure to eat	Healthy eating	0	
								Unhealthy eating	+	
							Restrictive Guidance	Healthy eating	0	
								Unhealthy eating	0	
4	Bante et al	2008	Journal of Nutrition Education and Behavior	Cross-sectional survey	1,555	US parents with child aged 2 to 5 living in rural areas	Reward	F&V preference	-	
								F&V intake	+	
							Pressure	F&V preference	-	
								F&V intake	-	
5	Berge et al	2010	Journal of Adolescent Health	Longitudinal survey	2,516	US children with a mean age of 12.8 (*SD* = 0.8) at beginning of study	Permissive Father	F&V intake	+ (later; girls)	
6	Boots et al	2015	Appetite	Cross-sectional survey	611	Australian parents of children aged 2 to 7 years old	(Control of) availability	Healthy snack intake	+	
								Unhealthy snack intake	-	
							Restrictive guidance	Healthy snack intake	-	
								Unhealthy snack intake	+	
							Demandingness	Healthy snack intake	+	
								Unhealthy snack intake	-	
							Responsiveness	Healthy snack intake	0	
								Unhealthy snack intake	+	

Table S1.

Study	Author(s)	Year	Outlet	Design	N	Participants	Independent Variable(s)	Dependent Variable (s)	Evidence of Effect	Moderators/ Mediators/ Comments
7	Brown et al	2008	Appetite	Cross-sectional survey	518	English parents with children aged 4 to 7	Restrictive guidance	Healthy snack intake	0	
								Unhealthy snack intake	0	
								F&V intake	+	
							(Control of) availability	Healthy snack intake	0	
								Unhealthy snack intake	-	
								F&V intake	+	
							Pressure to eat	Healthy snack intake	0	
								Unhealthy snack intake	+	
								F&V intake	-	
8	Brown & Ogden	2004	Health Education Research	Cross-sectional survey	112	British parent-child matched dyads with children aged 9 to 13	Modeling	Healthy snack intake	+	
								Unhealthy snack intake	+	
9	Brug et al	2008	British Journal of Nutrition	Cross-sectional survey	13,305	European children aged 8 to 14	Availability	F&V intake	+	
10	Campbell et al	2006	International Journal of Obesity	Cross-sectional survey	560	Australian parents with children aged 5 to 6	Modeling	F&V intake	+	
								Snack intake	0	
								High-energy drink intake	0	
							Pressure to eat	F&V intake	0	
								Snack intake	+	
								High-energy drink intake	+	
							Restrictive guidance	F&V intake	0	
								Snack intake	0	
								High-energy drink intake	0	
11	Cooke et al	2011	Psychological Science	Pre-test-post-test experimental design<br><br>3 treatment groups (exposure of food + non-food reward, exposure of food + social reward, exposure alone), 1 control group	422	British children aged 4 to 6	Reward	Vegetable liking	+	
								Vegetable intake	+	
							Reward (with praise)	Vegetable liking	+	
								Vegetable intake	+	
12	Cooke et al	2004	Public Health Nutrition	Cross-sectional survey	564	British parents of children aged 2 to 6	Modeling	F&V intake	+	

Table S1 (Continued)

Study	Author(s)	Year	Outlet	Design	N	Participants	Independent Variable(s)	Dependent Variable (s)	Evidence of Effect	Moderators/ Mediators/ Comments
13	Birch et al	1984	Child Development	3 (reward, praise, control) x 2 (single portion, double portion) experimental design	45	US children aged 3 to 5	Reward	Milk beverage preference	-	
							Reward (with praise)	Milk beverage preference	-	
14	Birch et al	1980	Child Development	4 (Food as reward, Food given without condition, food given in non-social context, food given during snack time) conditions over 4 time points	64	US children aged 3 to 6	Food as reward	Snack preference	+	
15	Campbell et al	2007	Obesity	Cross-sectional survey	347	Predominantly Australian mothers with children aged 12 to 13 years old	Modeling	High-energy drink intake	+	
								Sweet snack intake	0	
								Savory snack intake	+ (boys)	
							Pressure to eat	High-energy drink intake	0	
								Sweet snack intake	+ (boys)	
							Reward (with praise)	Savory snack intake	+	
							Authoritarian parenting style	High-energy drink intake	+ (boys)	
								Sweet snack intake	0	
							Availability	High-energy drink intake	+ (girls)	
								Sweet snack intake	+ (girls)	
								Savory snack	+	
16	Corsini et al	2013	Public Health Nutrition	Pre-test-post-test experimental design<br><br>2 treatment groups (exposure of food + reward, Exposure only), 1 control group	185	British children aged 4 to 6	Reward	Vegetable liking	+	
								Vegetable intake	+	
17	Cutler et al	2011	Journal of the American Dietetic Association	Longitudinal survey	4,746	US children aged 12 to 16	Availability	F&V intake	+	
								Snack intake	+	

Table S1 (Continued)

Study	Author(s)	Year	Outlet	Design	N	Participants	Independent Variable(s)	Dependent Variable (s)	Evidence of Effect	Moderators/ Mediators/Comments
18	Couch et al	2014	Journal of the Academy of Nutrition and Dietetics	Cross-sectional survey	699	US parent-child dyads with children aged 6 to 11	Modeling (2/5 items)	F&V intake	+	
								High-calorie beverages	0	
								Snacks	0	
							Restrictive guidance	F&V intake	0	
								High-calorie beverages	0	
								Snacks	0	
							Pressure to eat	F&V intake	0	
								High-calorie beverages	0	
								Snacks	0	
							Availability	F&V intake	0	
								High-calorie beverages	0	
								Snacks	+	
19	Cullen et al	2001	Health Education Research	Cross-sectional survey	230	US children aged 9 to 12	Availability	F&V intake	+	
							Modeling	F&V and juice intake	+	
20	De Bourdeaudhuij et al	2005	Public Health Nutrition	Cross-sectional survey	326	European children aged 10 to 11	Modeling	Fruits intake	+	
								Vegetables intake	+	
							Restrictive guidance	Fruits intake	+	
								Vegetables intake	+	
21	De Bourdeaudhuij et al	2009	Public Health Nutrition	Cross-sectional survey	4,555	European children aged 11	Parenting styles	F&V intake	0	Parenting styles moderated the impact of encouragement and restrictive guidance on fruit consumption and the impact of availability on fruits and vegetables
22	De Bourdeaudhuij et al	2008	European Journal of Clinical Nutrition	Cross-sectional survey	13,305	European children aged 11	Modeling	Fruits intake	+	Some differences across countries
								Vegetables intake	+	
							Availability	Fruits intake	0	
								Vegetables intake	+	
23	de Bruijn et al	2007	Health Education Research	Longitudinal survey	208	Dutch adolescent girls between 12 and 18	Restrictive guidance	SSB intake	-	Girls with moderate levels of agreeableness saw the most pronounced restrictive guidance effect on SSB consumption
								SSB subjective norms (to limit)	+	
								SSB attitudes	0	

Table S1 (Continued)

Study	Author(s)	Year	Outlet	Design	N	Participants	Independent Variable(s)	Dependent Variable (s)	Evidence of Effect	Moderators/ Mediators/Comments
24	Dickens & Ogden	2014	Appetite	Longitudinal survey	93	British parent-child dyads with children aged 17 and 18 at first wave (predominantly mothers)	Modeling	Unhealthy snacks	+<br>0	Note: children were living alone at follow-up
								Unhealthy meals	0	
								Healthy foods consumption	0	
							Restrictive guidance	Unhealthy snacks	0	
								Unhealthy meals	0	
								Healthy foods consumption	0	
							Availability	Unhealthy snacks	0	
								Unhealthy meals	0	
								Healthy foods consumption	0	
							Pressure to eat	Unhealthy snacks	0	
								Unhealthy meals	0	
								Healthy foods consumption		
25	Durao et al	2015	Appetite	Cross-sectional survey	4,122	Portuguese parents of children aged 4	Restrictive guidance	F&V intake (adequacy)	+	
								Snacks (over recommended)	-	
							(Control of) Availability	F&V intake (adequacy	+	
								Snacks (over recommended)	-	
							Pressure to eat	F&V intake (adequacy)	+	
								Snacks (over recommended)	+	
26	Eisenberg et al	2012	Journal of Nutrition Education and Behavior	Cross-sectional survey	541	US parents of children aged 5 to 8	Restrictive guidance and pressure to eat	Total fat consumption	+	
27	Elfhag et al	2008	Public Health Nutrition	Cross-sectional survey	1441	Swedish parent-child dyads with children aged 12	Modeling	F&V intake	+	
								SSB intake	+	
28	Fisher & Birch	2000	Journal of the American Dietetic Association	Quasi-experimental	197	US parent-child dyads with girls aged 4 to 6	Restrictive guidance and availability	Snacks intake	+	
29	Fisher & Birch	1999	Appetite	Quasi-experimental	71	US parent-child dyads with children aged 3 to 5	Restrictive guidance and availability	Snacks intake	+ (for girls)<br>0 (for boys)	
30	Fisher & Birch	1999	American Journal of Clinical Nutrition	Quasi-experimental	40	US parent-child dyads with children aged 3 to 6	Restrictive guidance and availability	Snacks intake	+	

Table S1 (Continued)

Study	Author(s)	Year	Outlet	Design	N	Participants	Independent Variable(s)	Dependent Variable (s)	Evidence of Effect	Moderators/ Mediators/Comments
31	Fisher et al	2002	Journal of the American Dietetic Association	Longitudinal survey	191	US parent-child dyads with girls aged 5	Pressure to eat	F&V intake	-	
							Modeling	F&V intake	+	
32	Gevers et al	2015	Appetite	Cross-sectional survey	329	Dutch children aged 11 to 15	Restrictive guidance	Snack intake	-	
33	Gibson et al	1998	Appetite	Cross-sectional survey	92	British mother-child dyads with children aged 9 to 11	Modeling	Fruits intake	+	
								Vegetables intake	0	
								Confectionary intake	0	
34	Entin et al	2014	Journal of the American College of Nutrition	Longitudinal survey	63	Israeli parent-child dyads with children aged 5 to 6; predominantly low SES	Availability	Vegetable intake	0	
								Fruits intake	0	
								Sweets & junk food intake	+	
							Modeling	Vegetable intake	+	
								Fruits intake	0	
								Sweets & junk food intake	0	
							Food as reward	Vegetable intake	0	
								Fruits intake	0	
								Sweets & junk food intake	+ (junk only)	
							Pressure to eat	Vegetable intake	0	
								Fruits intake	0	
								Sweets & junk food intake	+	
							Restrictive guidance	Vegetable intake	0	
								Fruits intake	0	
								Sweets & junk food intake	+	
35	Goldman et al	2012	Journal of Public Health Research	Cross-sectional survey	229	US parent-child dyads with children aged 2 to 5	Modeling	F&V intake	+	
							Availability	F&V intake	+	
							Accessibility	F&V intake	0	
36	Gross et al	2010	Journal of Nutrition Education and Behavior	Cross-sectional survey	93	US parent-child dyads predominantly mothers, with children aged 9 to 11	Modeling	F&V intake	0	
37	Gubbels et al	2009	Appetite	Cross-sectional survey	2,578	Dutch mothers with children aged 2	Restrictive guidance	Snacks, sweets and soft drinks	-	
								F&V intake	+	

Table S1 (Continued)

Study	Author(s)	Year	Outlet	Design	N	Participants	Independent Variable(s)	Dependent Variable (s)	Evidence of Effect	Moderators/ Mediators/Comments
38	Gregory et al	2011	Appetite	Longitudinal survey	60	Australian mothers of children aged 2 to 4	Restrictive guidance	Fruit consumption	0	Results are controlled for consumption at first wave of study; correlational analysis at baseline revealed greater number significant findings
								Vegetables consumption	0	
								Sweets consumption	0	
							Pressure to eat	Fruit consumption	-	
								Vegetables consumption	-	
								Sweets consumption	0	
							Modeling	Fruit consumption	0	
								Vegetables consumption	+	
								Sweets consumption	0	
							Availability	Fruit consumption	0	
								Vegetables consumption	0	
								Sweets consumption	0	
39	Harris & Ramsey	2015	Appetite	Cross-sectional survey	102	US African American fathers with children aged 3 to 13	Modeling	F&V intake	+	
								SSBs intake	+	
							Availability of healthy food	F&V intake	0	
								SSBs intake	-	
40	Hendy et al	2009	Appetite	Cross-sectional survey	1499	US mothers with children aged 6 to 11	Restrictive guidance	Snacks intake	0	
							Availability of fruits and veg	F&V intake	+	
							Reward	Snacks intake	0	
								F&V intake	0	
							Pressure to eat	Snacks intake	0	
								F&V intake	0	
							Modeling (of snacks)	Snacks intake	+	

Table S1 (Continued)

Study	Author(s)	Year	Outlet	Design	N	Participants	Independent Variable(s)	Dependent Variable (s)	Evidence of Effect	Moderators/ Mediators/Comments
41	Hennessy et al	2012	Journal of the American Dietetic Association	Cross-sectional survey	99	US parent-child dyads with children aged 6 to 11 living in rural areas; predominantly mothers	Authoritarian parental *feeding* style<br>Uninvolved parental *feeding* style<br>Permissive parental *feeding* style<br>Restrictive guidance<br>Pressure to eat	Low-nutrient-dense foods intake (LNDF)<br>LNDF intake<br>LNDF intake<br>LNDF intake<br>LNDF intake	0<br>0<br>+<br>0<br>0	Moderating effect of feeding styles on restrictive guidance was found; specifically, restrictive guidance led to lower intake of LNDF among unpermissive parents<br><br>Other moderating effects of parental feeding styles were not found
42	Hoerr et al	2009	International Journal of Behavioral Nutrition and Physical Activity	Cross-sectional survey	715	US parent-child dyads with children aged 3 to 5; predominantly mothers, and low-income households	Authoritarian parental *feeding* styles	Fruits, juice and vegetables intake	-	In comparison to permissive parental *feeding* styles
43	Jansen et al	2007	Appetite	Pre-test-post-test experimental design<br><br>2 (prohibition/no prohibition)x2 (phase1/phase2)	74	European children aged 5 to 6	Restrictive guidance (of experimenter)<br><br>Restrictive guidance (of parents)	Snacks desire<br>Snacks intake<br><br>Snacks intake	+<br>+ (larger proportion consumed)<br>Curvilinear	Children with parents of low restrictive guidance and high restrictive guidance consumed more food than those with moderate restrictive guidance
44	Johnson et al	2011	British Journal of Nutrition	Cross-sectional survey	342	UK parent-child sample of twins aged 11	Maternal Modeling<br><br>Paternal Modeling<br><br>Availability<br><br>Restrictive guidance<br><br>Pressure to eat	Core food intake<br>Non-core food intake<br>Core food intake<br>Non-core food intake<br>Core food intake<br>Non-core food intake<br>Core food intake<br>Non-core food intake<br>Core food intake<br>Non-core food intake	+<br>+<br>0<br>0<br>0<br>+<br>0<br>0<br>0<br>0	
45	Kiefner-Burmeister et al	2014	Appetite	Cross-sectional survey	171	US mothers of children aged 3 to 6	Food as reward, as emotion regulation, and restrictive guidance (measured in reverse)	F&V intake<br>Snack, sweets, and SSBs intake	-<br>+	The latent variable of “negative feeding practices” combined these different dimensions of parenting practices

Table S1 (Continued)

Study	Author(s)	Year	Outlet	Design	N	Participants	Independent Variable(s)	Dependent Variable (s)	Evidence of Effect	Moderators/ Mediators/Comments
46	Koui & Jago	2008	Public Health Nutrition	Cross-sectional survey	167	Greek children aged 10 to 12	Availability	F&V intake	+	
47	Kremers, Brug, de Vries, & Engels	2003	Appetite	Cross-sectional survey	643	Dutch children aged 16 to 17	Authoritative parenting style	Fruits attitude	+	Analyses based on ANOVA and evidence of effects are based on mean comparison with children classified under other parenting styles
								Fruits subjective norm	+	
								Fruit intention to eat	+	
								Fruits intake	+	
							Indulgent parenting style	Fruits attitude	+	
48	Kröller & Warschburger	2009	International Journal of Behavioral Nutrition and Physical Activity	Cross-sectional survey	556	German mothers of children aged 1 to 10	Restrictive guidance	Unhealthy food intake	0	
								Healthy food intake	0	
							Pressure to eat	Unhealthy food intake	0	
								Healthy food intake	0	
							Food as reward	Unhealthy food intake	+	
								Healthy food intake	0	
							Modeling (as intentional act of eating healthy in front of children)	Unhealthy food intake	-	
								Healthy food intake	+	
49	Lee & Keller	2012	Journal of the Academy of Nutrition and Dietetics	Cross-sectional design with lab-based measure of food consumption	68	US children aged 4 to 6	Pressure to eat (measured outside of lab)	Mac and cheese intake	-	
								Vegetables intake	+	
								Fruits intake	0	
								Milk intake	+	
								SSBs intake	0	
								Chocolate pudding intake	-	
50	Lo et al	2015	PLoS ONE	Cross-sectional survey	4,553	Hong Kong parents of children aged 2 to 5	Food as reward	High energy-density food	+	
								Fruits intake	0	
								Vegetables intake	-	
							Encouragement and rewarding with praise	High energy-density food	0	
								Fruits intake	+	
								Vegetables intake	+	
							Restrictive guidance	High energy-density food	-<br>0	
								Fruits intake	+	
								Vegetables intake		

Table S1 (Continued)

Study	Author(s)	Year	Outlet	Design	N	Participants	Independent Variable(s)	Dependent Variable (s)	Evidence of Effect	Moderators/ Mediators/Comments
51	Lopez et al	2012	Journal of the Academy of Nutrition and Dietetics	Cross-sectional survey	541	US parents of children aged 5 to 8	Restrictive guidance	SSBs intake	0	
52	Loth et al	2016	Appetite	Cross-sectional survey	2383	US parent-child dyads with children with a mean age of 14.4 (*SD* = 2.0)	Availability and accessibility of healthy food	F&V intake<br>Snacks intake<br>SSBs intake	+<br>-<br>-	
							Healthy food modeling	F&V intake<br>Snacks intake<br>SSBs intake	+<br>-<br>-	
							Restrictive guidance	F&V intake<br>Snacks intake<br>SSBs intake	+<br>+<br>0	
53	McGowan et al	2012	European Journal of Clinical Nutrition	Cross-sectional survey	434	UK primary caregivers of children aged 2 to 5	Modeling	F&V intake<br>Snacks intake<br>Non-core drinks intake	+<br>+<br>+	
							Availability	F&V intake<br>Snacks intake<br>Non-core drinks intake	0<br>+<br>0	
54	Monge-Rojas et al	2010	Appetite	Cross-sectional survey	133	Costa Rican children aged 14 to 18	Restrictive guidance	Fruits intake<br>Vegetables intake<br>Fast food intake	0<br>0<br>-	
55	Østbye et al	2013	International Journal of Obesity	Cross-sectional study	208	US mothers of children aged 2 to 5	Availability (of junk food)	Junk food intake<br>Healthy food intake	+<br>-	
							Modeling (of healthy eating)	Junk food intake<br>Healthy food intake	-<br>+	
56	Nickelson et al	2012	Journal of Nutrition Education and Behavior	Cross-sectional survey	4,049	US children aged 11 to 14	Restrictive guidance	SSBs purchase and intake	-	

Table S1 (Continued)

Study	Author(s)	Year	Outlet	Design	N	Participants	Independent Variable(s)	Dependent Variable (s)	Evidence of Effect	Moderators/ Mediators/Comments
57	McPhie et al	2012	Early Child Development and Care	Longitudinal survey	117	Australian parents with children aged 2 to 4	Restrictive guidance	Fruits intake	+	DVs are collected at second wave of data collection
								Vegetables intake	0	
								Unhealthy food intake	0	
							Pressure to eat	F&V intake	0	
								Unhealthy food intake	0	
							Maternal warmth	F&V intake	0	
								Unhealthy food intake	-	
								F&V intake	0	
							Maternal control	Unhealthy food intake	0	
58	Melbye & Hansen	2015	BioMed Research International	Cross-sectional survey	796	Norwegian parent-child dyads with children aged 10 to 12	Active guidance	Vegetables intake	+	
								SSBs intake	-	
							Availability of healthy food	Vegetables intake	+	
								SSBs intake	-	
59	Melbye et al	2013	Appetite	Cross-sectional survey (dataset corresponds with previous study)	796	Norwegian parent-child dyads with children aged 10 to 12	Availability of healthy foods	Vegetables intake	+	Self-efficacy mediated the relationship between restrictive guidance and vegetables intake<br><br>Differences in results with Study 60 is a consequence of different analytical procedure used (this study utilized regressions, while Study 60 utilized correlations)<br><br>In another study with the same dataset [60], the same IVs here had no effect on fruits intake.
							Restrictive guidance	Vegetables intake	-	
							Active guidance	Vegetables intake	0	
							Modeling	Vegetables intake	0	
							Food as reward	Vegetables intake	0	
60	Palfreyman et al	2014	Maternal and Child Nutrition	Cross-sectional survey	484	British mothers with children aged 18 months to 8 years old	Modeling (analyzed as 3 dimensions)	F&V intake	+	Modeling is conceptualized as having 3 dimensions in this study. The effects listed here assume any one dimension's impact on consumption behavior as indicative of a significant modeling effect.
								Sweet snacks intake	0	
								Savory snacks intake	+	
								Rice, potatoes, pasta intake	0	
								Salads intake	+	
								Fresh fruit juice intake	0	

Table S1 (Continued)

Study	Author(s)	Year	Outlet	Design	N	Participants	Independent Variable(s)	Dependent Variable (s)	Evidence of Effect	Moderators/ Mediators/Comments
61	Papaioannou et al	2013	Journal of Nutrition Education and Behavior	Cross-sectional survey	667	Predominantly US mothers with children with a mean age of 4.4 (*SD* = 0.6)	Active guidance	F&V Intake	0	Indulgent parental *feeding* style moderated the effect of restrictive guidance on fruit and vegetable intake; among indulgent parents, restrictive guidance saw stronger associations with fruits and vegetables intake
							Restrictive guidance	F&V Intake	0	
							Practical methods (includes reward, food as reward, reward with praise)	F&V Intake	0	
							Availability (of healthy food)	F&V Intake	0	
62	Patrick et al	2005	Appetite	Cross-sectional survey	231	US caregivers of children aged 3 to 5; predominantly women	Authoritative feeding style	Fruits intake	0	
								Vegetables intake	+	
								Dairy intake	+	
							Authoritarian feeding style	Fruits intake	0	
								Vegetables intake	-	
								Dairy intake	0	
63	Pearson et al	2009	Public Health Nutrition	Cross-sectional study	328	British children aged 12 to 16	Authoritative parenting	Fruits intake	+	
								Unhealthy snacks intake	-	
								Vegetables intake	0	
64	Peters et al	2012	Public Health Nutrition	Cross-sectional study	269	Australian parents with children aged 2 to 5	Parenting styles	F&V intake	0	
								Non-core foods intake	0	
							Pressure to eat	F&V intake	0	
								Non-core foods intake	0	
							Restrictive guidance	F&V intake	-	
								Non-core foods intake	0	
65	Ray et al	2013	Journal of Nutrition Education and Behavior	Cross-sectional study	805	Finnish parent-child dyads with children aged 10 to 11	Restrictive guidance (only 1 out 3 items in their measure is indicative of our construct; however, this study was added as it is an illustration of a moderating effect of parenting styles)	Nutrient-dense foods intake	+	Parental warmth moderates the association between restrictive guidance and nutrient-dense and energy-dense foods intake, such that there are stronger associations with nutrient-dense foods intake when parental warmth is high, while there are stronger associations with energy-dense foods when parental warmth is low
								Energy-dense foods intake	-	

Table S1 (Continued)

Study	Author(s)	Year	Outlet	Design	N	Participants	Independent Variable(s)	Dependent Variable (s)	Evidence of Effect	Moderators/ Mediators/Comments
66	Ray et al	2013	Public Health Nutrition	Cross-sectional study	8,736	European children aged 10 to 12	Modeling	Fruits intake	+	
								Vegetables intake	+	
							Accessibility	Fruits intake	+	
								Vegetables intake	+	
67	Reinaerts et al	2007	Appetite	Cross-sectional survey	1,739	Dutch parents of children aged 4 to 12	Modeling	Fruits intake	+	
								Vegetables intake	+	
							Accessibility	Fruits intake	0	
							Availability	Fruits intake	+	
								Vegetables intake	0	
68	Rodenburg et al	2014	Public Health Nutrition	Longitudinal survey	1,275	Dutch parent-child dyads with children aged 9	Food as reward	Fruits intake	-	Psychological and behavioral control moderated the effects of food as reward on fruit intake (high = significant association), the effects of availability (of healthy food) on snacking (low = significant association), and the effects of restrictive guidance on SSBs intake (low = significant association)
								Snacks intake	+	
								SSBs intake	0	
							Restrictive guidance	Fruits intake	0	
								Snacks intake	-	
								SSBs intake	-	
							Availability (of healthy food)	Fruits intake	+	
								Snacks intake	-	
								SSBs intake	-	
69	Rodenburg et al	2012	Appetite	Cross-sectional survey	1,762	Dutch parent-child dyads with children aged 8	Modeling	Fruits intake	+	Psychological and behavioral control moderated the effects of modeling. High control parent-child relationship displayed strongest associations between modeling and child intake
70	Schwartz et al	2015	Childhood Obesity	Longitudinal survey	480	US children aged 11 to 12 at first wave of data collection, predominantly low-income, black, and Latino.	Authoritative parenting	SSBs intake	-	Television in child's room moderates the influence of authoritative parenting on SSBs intake by weakening its association
71	Sdrali et al	2010	International Journal of Consumer Studies	Cross-sectional survey	149	Greek children aged 13 to 16	Availability, accessibility, modeling, injunctive and descriptive norms	SSBs intake	+	

Table S1 (Continued)

Study	Author(s)	Year	Outlet	Design	N	Participants	Independent Variable(s)	Dependent Variable (s)	Evidence of Effect	Moderators/ Mediators/Comments
72	Sleddens et al	2010	Appetite	Cross-sectional survey	135	Dutch parents of children aged 6 to 7	Food as reward	Snacks intake	0	
								Fruits intake	0	
								SSBs intake	0	
							Restrictive guidance	Snacks intake	0	
								Fruits intake	0	
								SSBs intake	0	
73	Sleddens et al	2014	Appetite	Longitudinal survey	1654	Dutch parents of children aged 6 at first time point, and 8 at second time point	Food as reward	Snacks intake	0	General parenting styles as measured by the Comprehensive General Parenting Questionnaire moderated the effects of some parenting practices on eating. Manipulating availability and restrictive guidance had stronger effects on children in a positive (less overprotection and controlling) parenting context.
								SSBs intake	0	
								Water intake	0	
								Fruits intake	-	
							Restrictive guidance	Snacks intake	0	
								SSBs intake	-	
								Water intake	+	
								Fruits intake	0	
							Availability (healthy foods)	Snacks intake	-	
								SSBs intake	-	
								Water intake	+	
								Fruits intake	+	
							Pressure to eat	Snacks intake	+	
								SSBs intake	+	
								Water intake	0	
								Fruits intake	0	
74	van Ansem et al	2014	British Journal of Nutrition	Cross-sectional survey	1428	Dutch parents of children aged 8 to 12	Modeling	SSBs intake	+	
							Restrictive guidance	SSBs intake	0	
							Availability	SSBs intake	+	
75	van Grieken et al	2015	Pediatric Obesity	Longitudinal survey	2047	Dutch parent-child dyads with children aged 5 at first wave; 7 at second wave	Restrictive guidance	SSBs intake	-	
							Availability (healthy foods)	SSBs intake	-	
76	Van Lippevelde et al	2013	Appetite	Cross-sectional survey	6512	European parent-child dyads with children with a mean age of 11.7 (*SD* = 0.8)	Active guidance	SSBs intake	0	
								Juice intake	0	
							Availability	SSBs intake	+	
								Juice intake	+	
							Modeling	SSBs intake	+	
								Juice intake	+	
							Food as reward	SSBs intake	0	
								Juice intake	0	

Table S1 (Continued)

Study	Author(s)	Year	Outlet	Design	N	Participants	Independent Variable(s)	Dependent Variable (s)	Evidence of Effect	Moderators/ Mediators/Comments
77	Taylor et al	2011	Appetite	Cross-sectional survey	175	Australian caregivers of children with a mean age of 9.21 (*SD* = 1.09)	Parent-report parenting style	F&V intake	0	Parenting styles did not interact with parenting practices in predicting food consumption-related variables
								Non-core food	0	
								intake	0	
								Attitude (veg)	0	
								Attitude (fruit)	0	
								Attitude (non-core food)	0	
							Child-reported responsiveness	F&V intake	0	
								Non-core food	0	
								intake	0	
								Attitude (veg)	0	
								Attitude (fruit)	+	
								Attitude (non-core food)	-	
							Child-reported demandingness	F&V intake	0	
								Non-core food	0	
								intake	0	
								Attitude (veg)	0	
								Attitude (fruit)	+	
								Attitude (non-core food)	0	
							Restrictive guidance	F&V intake	0	
								Non-core food	0	
								intake	0	
								Attitude (veg)	0	
								Attitude (fruit)	0	
								Attitude (non-core food)	0	
							Pressure to eat	F&V intake	0	
								Non-core food	0	
								intake	0	
								Attitude (veg)	0	
								Attitude (fruit)	0	
								Attitude (non-core	0	
								food)	0	
78	van der Horst et al	2007	Health Education Research	Cross-sectional survey	383	Dutch children aged 12 to 17	Restrictive guidance and availability (with availability reverse-coded)	SSBs intake	-	Attitude, self-efficacy, habit strength mediated the relationship between restrictive guidance + availability on SSBs intake
							Modeling	SSBs intake	+	Parenting styles moderated the relationship between restrictive guidance + availability on SSBs intake; more effective among highly involved, as well as moderately strict parents

Table S1 (Continued)

Study	Author(s)	Year	Outlet	Design	N	Participants	Independent Variable(s)	Dependent Variable (s)	Evidence of Effect	Moderators/ Mediators/Comments
79	Van Strien et al	2009	Appetite	Cross-sectional survey	943	Dutch children aged 7 to 12	Restrictive guidance	Snacking	-	Bivariate correlations showed more significant correlations
								Fruits intake	+	
							Pressure to eat	Snacking	0	
								Fruits intake	0	
80	Vereecken et al	2004	Appetite	Cross-sectional survey	346	Dutch mothers of children aged 2 to 7	Restrictive guidance	Fruits intake	0	
								Vegetables intake	0	
								SSBs intake	-	
								Sweets intake	-	
							Active guidance	Fruits intake	0	
								Vegetables intake	0	
								SSBs intake	0	
								Sweets intake	0	
							Pressure to eat	Fruits intake	0	
								Vegetables intake	0	
								SSBs intake	0	
								Sweets intake	0	
							Reward	Fruits intake	0	
								Vegetables intake	0	
								SSBs intake	0	
								Sweets intake	+	
							Reward with praise	Fruits intake	0	
								Vegetables intake	+	
								SSBs intake	0	
								Sweets intake	0	
							Avoid negative modeling	Fruits intake	0	
								Vegetables intake	0	
								SSBs intake	0	
								Sweets intake	0	
							Modeling	Fruits intake	+	
								Vegetables intake	+	
								SSBs intake	+	
								Sweets intake	+	
81	Wardle et al	2005	Journal of the American Dietetic Association	Cross-sectional survey	564	British parents of children aged 2 to 6	Pressure to eat	F&V intake	-	
							Modeling	F&V intake	+	

Table S1 (Continued)

Study	Author(s)	Year	Outlet	Design	N	Participants	Independent Variable(s)	Dependent Variable (s)	Evidence of Effect	Moderators/ Mediators/Comments
82	Vereecken et al	2010	Public Health Nutrition	Longitudinal study	609	Belgian mother-child dyads with children aged 9 to 11 at first wave	Restrictive guidance	F&V intake	0	Bivariate correlations showed more significant correlations
								SSBs and snacks intake	-	
							Pressure to eat	F&V intake	0	
								SSBs and snacks intake	0	
							Reward	F&V intake	0	
								SSBs and snacks intake	0	
							Reward with praise	F&V intake	0	
								SSBs and snacks intake	0	
							Availability (of unhealthy food)	F&V intake	-	
								SSBs and snacks intake	+	
							Avoid negative modeling	F&V intake	0	
								SSBs and snacks intake	0	
							Modeling	F&V intake	+	
								SSBs and snacks intake	+	
83	Vereecken et al	2009	American Journal of Health Promotion	Cross-sectional study	1614	Belgian parent-child dyads with children aged 11 to 12	Restrictive guidance	Fruits intake	+	
								Vegetables intake	+	
								SSBs intake	-	
								Sweets intake	-	
							Pressure to eat	Fruits intake	-	
								Vegetables intake	0	
								SSBs intake	0	
								Sweets intake	+	
							Reward	Fruits intake	0	
								Vegetables intake	0	
								SSBs intake	0	
								Sweets intake	0	
							Reward with praise	Fruits intake	0	
								Vegetables intake	0	
								SSBs intake	0	
								Sweets intake	0	
							Avoid negative modeling	Fruits intake	0	
								Vegetables intake	0	
								SSBs intake	0	
								Sweets intake	0	
							Parenting styles	Fruits intake	0	
								Vegetables intake	0	
								SSBs intake	0	
								Sweets intake	0	
84	Vereecken et al	2010	Appetite	Cross-sectional study	755	Belgian parents of children with a mean age of 3.5	Modeling	F&V intake	+	
							Parenting styles (laxness, support, overreactivity)	F&V intake	0	

Table S1 (Continued)

Study	Author(s)	Year	Outlet	Design	N	Participants	Independent Variable(s)	Dependent Variable (s)	Evidence of Effect	Moderators/ Mediators/Comments
85	Wyse et al	2011	BMC Public Health	Cross-sectional survey	396	Australian parents of children aged 3 to 5	Modeling	F&V intake	+	
							Pressure to eat	F&V intake	0	
							Availability	F&V intake	+	
							Accessibility	F&V intake	+	
							Food as reward	F&V intake	0	
							Restrictive guidance	F&V intake	0	
86	Xu et al	2013	Appetite	Cross-sectional survey	242	Australian mother with children aged 2	Parental warmth	Fruits intake	0	
								Vegetables intake	+	
								SSBs intake	0	
								Snacks intake	0	
							Parental hostility	Fruits intake	-	
								Vegetables intake	0	
								SSBs intake	+	
								Snacks intake	+	
87	Young et al	2004	Journal of Nutrition Education and Behavior	Cross sectional survey	366	US children aged 12 to 16	Authoritative parenting	F&V intake	0	Self-efficacy partially mediated the effect of modeling on F&V intake
							Restrictive guidance	F&V intake	0	
							Modeling	F&V intake	+	Availability moderated the effect of modeling on F&V intake such that the effect of modeling is stronger when there is higher availability of F&V
							Availability	F&V intake	+	
88	Zahra et al	2014	Chid: Care, Health and Development	Cross-sectional survey	10,645	British children aged 12 to 16	Authoritative parenting	Junk food intake	-	

Table S1 (Continued)

References

1. Alsharairi NA, Somerset SM. Associations between parenting styles and children's fruit and vegetable intake. Ecol. Food Nutr. [Internet]. 2015;54:93–113. Available from: <Go to ISI>://WOS:000347520800007

2. Amuta AO, Jacobs W, Idoko EE, Barry AE, McKyer ELJ. Influence of the home food environment on children's fruit and vegetable consumption: A study of rural low-income families. Health Promot. Pract. [Internet]. 2015;16:689–98. Available from: <Go to ISI>://WOS:000359922200008

3. Arredondo EM, Elder JP, Ayala GX, Campbell N, Baquero B, Duerksen S. Is parenting style related to children's healthy eating and physical activity in Latino families? Health Educ. Res. [Internet]. 2006;21:862–71. Available from: <Go to ISI>://WOS:000242473500012

4. Bante H, Elliott M, Harrod A, Haire-Joshu D. The use of inappropriate feeding practices by rural parents and their effect on preschoolers' fruit and vegetable preferences and intake. J. Nutr. Educ. Behav. [Internet]. Elsevier. 6277 Sea Harbor Drive, Orlando, FL 32887-4800.; 2008;40:28–33. Available from: http://search.proquest.com/docview/61965345?accountid=12665

5. Berge JM, Wall M, Loth KA, Neumark-Sztainer D. Parenting style as a predictor of adolescent weight and weight-related behaviors. J. Adolesc. Heal. [Internet]. 2010;46:331–8. Available from: <Go to ISI>://WOS:000275784700005

6. Boots SB, Tiggemann M, Corsini N, Mattiske J. Managing young children's snack food intake. The role of parenting style and feeding strategies. Appetite [Internet]. 2015;92:94–101. Available from: <Go to ISI>://WOS:000358269100014

7. Brown KA, Ogden J, Vögele C, Gibson EL. The role of parental control practices in explaining children's diet and BMI. Appetite [Internet]. Netherlands: Elsevier Science; 2008;50:252–9. Available from: http://search.ebscohost.com/login.aspx?direct=true&db=psyh&AN=2008-02994-009&site=ehost-live

8. Brown R, Ogden J. Children's eating attitudes and behaviour: A study of the modelling and control theories of parental influence. Health Educ. Res. [Internet]. Oxford University Press. Great Clarendon Street, Oxford OX2 6DP, UK. Tel: +44-1865-353907; Fax: +44-1865-353485; e-mail: jnls.cust.serv@oxfordjournals.org; Web site: http://her.oxfordjournals.org/; 2004;19:261–71. Available from: http://search.proquest.com/docview/1011398496?accountid=12665

9. Brug J, Tak NI, Velde SJ te, Bere E, de Bourdeaudhuij I. Taste preferences, liking and other factors related to fruit and vegetable intakes among schoolchildren: Results from observational studies. Br. J. Nutr. [Internet]. 2008;99:S7–14. Available from: <Go to ISI>://WOS:000253679700003

10. Campbell KJ, Crawford DA, Ball K. Family food environment and dietary behaviors likely to promote fatness in 5-6 year-old children. Int. J. Obes. 2006/02/24 ed. 2006;30:1272–80.

11. Cooke LJ, Chambers LC, Anez E V, Croker HA, Boniface D, Yeomans MR, et al. Eating for pleasure or profit: The effect of incentives on children's enjoyment of vegetables. Psychol. Sci. 2010/12/31 ed. 2011;22:190–6.

12. Cooke LJ, Wardle J, Gibson EL, Sapochnik M, Sheiham A, Lawson M. Demographic, familial and trait predictors of fruit and vegetable consumption by pre-school children. Public Health Nutr. [Internet]. 2004;7:295–302. Available from: <Go to ISI>://WOS:000220567200008

13. Birch LL, Marlin DW, Rotter J. Eating as the "means" activity in a contingency: Effects on young children's food preference. Child Dev. [Internet]. 1984;55:431. Available from: http://www.jstor.org/stable/1129954?origin=crossref

14. Birch LL, Zimmerman SI, Hind H. The influence of social-affective context on the formation of children's food preferences. Child Dev. 1980;51:856–61.

15. Campbell KJ, Crawford DA, Salmon J, Carver A, Garnett SP, Baur LA. Associations between the home food environment and obesity-promoting eating behaviors in adolescence. Obes. (Silver Spring). 2007/03/21 ed. 2007;15:719–30.

16. Corsini N, Slater A, Harrison A, Cooke L, Cox DN. Rewards can be used effectively with repeated exposure to increase liking of vegetables in 4-6-year-old children. Public Health Nutr. 2011/09/09 ed. 2013;16:942–51.

17. Cutler GJ, Flood A, Hannan P, Neumark-Sztainer D. Multiple sociodemographic and socioenvironmental characteristics are correlated with major patterns of dietary intake in adolescents. J. Am. Diet. Assoc. 2011/01/29 ed. 2011;111:230–40.

18. Couch SC, Glanz K, Zhou C, Sallis JF, Saelens BE. Home food environment in relation to children's diet quality and weight status. J. Acad. Nutr. Diet. [Internet]. 2014;114:1569–79. Available from: <Go to ISI>://WOS:000342610700009

19. Cullen KW, Baranowski T, Rittenberry L, Cosart C, Hebert D, de Moor C. Child-reported family and peer influences on fruit, juice and vegetable consumption: Reliability and validity of measures. Health Educ. Res. [Internet]. United Kingdom: Oxford University Press; 2001;16:187–200. Available from: http://search.ebscohost.com/login.aspx?direct=true&db=psyh&AN=2001-17128-006&site=ehost-live

20. De Bourdeaudhuij I, Klepp K-I, Due P, Rodrigo CP, de Almeida M, Wind M, et al. Reliability and validity of a questionnaire to measure personal, social and environmental correlates of fruit and vegetable intake in 10–11-year-old children in five European countries. Public Health Nutr. [Internet]. 2005;8. Available from: http://www.journals.cambridge.org/abstract_S1368980005000261

21. De Bourdeaudhuij I, te Velde SJ, Maes L, Perez-Rodrigo C, de Almeida MD V, Brug J. General parenting styles are not strongly associated with fruit and vegetable intake and social-environmental correlates among 11-year-old children in four countries in Europe. Public Health Nutr. [Internet]. 2009;12:259–66. Available from: <Go to ISI>://WOS:000263226700017

22. De Bourdeaudhuij I, te Velde S, Brug J, Due P, Wind M, Sandvik C, et al. Personal, social and environmental predictors of daily fruit and vegetable intake in 11-year-old children in nine European countries. Eur. J. Clin. Nutr. [Internet]. 2007/05/25 ed. 2008;62:834–41. Available from: http://www.nature.com.ezproxy.library.wur.nl/ejcn/journal/v62/n7/abs/1602794a.html

23. de Bruijn GJ, Kremers SPJ, de Vries H, van Mechelen W, Brug J. Associations of social-environmental and individual-level factors with adolescent soft drink consumption: Results

from the SMILE study. Heal. Educ. Res. Res. 2006/08/02 ed. 2007;22:227–37.

24. Dickens E, Ogden J. The role of parental control and modelling in predicting a child's diet and relationship with food after they leave home. A prospective study. Appetite. 2014/02/01 ed. 2014;76:23–9.

25. Durao C, Andreozzi V, Oliveira A, Moreira P, Guerra A, Barros H, et al. Maternal child-feeding practices and dietary inadequacy of 4-year-old children. Appetite [Internet]. 2015;92:15–23. Available from: <Go to ISI>://WOS:000358269100003

26. Eisenberg CM, Ayala GX, Crespo NC, Lopez N V, Zive MM, Corder K, et al. Examining multiple parenting behaviors on young children's dietary fat consumption. J. Nutr. Educ. Behav. [Internet]. 2012;44:302–9. Available from: <Go to ISI>://WOS:000306066000005

27. Elfhag K, Tholin S, Rasmussen F. Consumption of fruit, vegetables, sweets and soft drinks are associated with psychological dimensions of eating behaviour in parents and their 12-year-old children. Public Health Nutr. [Internet]. 2008;11:914–23. Available from: http://www.ncbi.nlm.nih.gov/pubmed/18498675

28. Fisher JO, Birch LL. Parents' restrictive feeding practices are associated with young girls' negative self-evaluation of eating. J. Am. Diet. Assoc. 2000/12/05 ed. 2000;100:1341–6.

29. Fisher JO, Birch LL. Restricting access to foods and children's eating. Appetite. 1999/05/25 ed. 1999;32:405–19.

30. Fisher JO, Birch LL. Restricting access to a palatable food affects children's behavioral response, food selection and intake. Am. J. Clin. Nutr. 1999;69:1264–72.

31. Fisher JO, Mitchell DC, Smiciklas-Wright H, Birch LL. Parental influences on young girls' fruit and vegetable, micronutrient, and fat intakes. J. Am. Diet. Assoc. Department of Pediatrics, US Department of Agriculture Children's Nutrition Research Center, Baylor College of Medicine, Houston, Texas, USA. SRC - Pubmed ID2 - 11794503 FG - 0; 2002;102:58–64.

32. Gevers DWM, van Assema P, Sleddens EFC, de Vries NK, Kremers SPJ. Associations between general parenting, restrictive snacking rules, and adolescent's snack intake. The roles of fathers and mothers and interparental congruence. Appetite [Internet]. 2015;87:184–91. Available from: <Go to ISI>://WOS:000350517800023

33. Gibson EL, Wardle J, Watts CJ. Fruit and vegetable consumption, nutritional knowledge and beliefs in mothers and children. Appetite. 1998/10/30 ed. 1998;31:205–28.

34. Entin A, Kaufman-Shriqui V, Naggan L, Vardi H, Shahar DR. Parental feeding practices in relation to low diet quality and obesity among LSES children. J. Am. Coll. Nutr. [Internet]. 2014;33:306–14. Available from: <Go to ISI>://WOS:000342139900007

35. Goldman RL, Radnitz CL, McGrath RE. The role of family variables in fruit and vegetable consumption in pre-school children. J. Public health Res. 2012/06/15 ed. 2012;1:143–8.

36. Gross SM, Pollock ED, Braun B. Family influence: Key to fruit and vegetable consumption among fourth- and fifth-grade students. J. Nutr. Educ. Behav. 2010/05/11 ed. 2010;42:235–41.

37. Gubbels JS, Kremers SPJ, Stafleu A, Dagnelie PC, Goldbohm RA, de Vries NK, et al. Diet-related restrictive parenting practices. Impact on dietary intake of 2-year-old children

and interactions with child characteristics. Appetite [Internet]. 2009;52:423–9. Available from: <Go to ISI>://WOS:000264632600022

38. Gregory JE, Paxton SJ, Brozovic AM. Maternal feeding practices, child eating behaviour and body mass index in preschool-aged children: a prospective analysis. Int. J. Behav. Nutr. Phys. Act. [Internet]. 2010;7. Available from: <Go to ISI>://WOS:000280403900001

39. Harris TS, Ramsey M. Paternal modeling, household availability, and paternal intake as predictors of fruit, vegetable, and sweetened beverage consumption among African American children. Appetite [Internet]. 2015;85:171–7. Available from: <Go to ISI>://WOS:000347867000024

40. Hendy HM, Williams KE, Camise TS, Eckman N, Hedemann A. The Parent Mealtime Action Scale (PMAS). Development and association with children's diet and weight. Appetite [Internet]. 2009;52:328–39. Available from: <Go to ISI>://WOS:000264632600010

41. Hennessy E, Hughes SO, Goldberg JP, Hyatt RR, Economos CD. Parent behavior and child weight status among a diverse group of underserved rural families. Appetite [Internet]. 2010;54:369–77. Available from: <Go to ISI>://WOS:000276005100018

42. Hoerr SL, Hughes SO, Fisher JO, Nicklas TA, Liu Y, Shewchuk RM. Associations among parental feeding styles and children's food intake in families with limited incomes. Int. J. Behav. Nutr. Phys. Act. [Internet]. 2009;6. Available from: <Go to ISI>://WOS:000270481700001

43. Jansen E, Mulkens S, Jansen A. Do not eat the red food!: Prohibition of snacks leads to their relatively higher consumption in children. Appetite. 2007;49:572–7.

44. Johnson L, van Jaarsveld CHM, Wardle J. Individual and family environment correlates differ for consumption of core and non-core foods in children. Br. J. Nutr. [Internet]. 2011;105:950–9. Available from: <Go to ISI>://WOS:000288131000017

45. Kiefner-Burmeister AE, Hoffmann DA, Meers MR, Koball AM, Musher-Eizenman DR. Food consumption by young children: A function of parental feeding goals and practices. Appetite [Internet]. 2014;74:6–11. Available from: <Go to ISI>://WOS:000331481800002

46. Koui E, Jago R. Associations between self-reported fruit and vegetable consumption and home availability of fruit and vegetables among Greek primary-school children. Public Health Nutr. [Internet]. 2008;11:1142–8. Available from: http://www.ncbi.nlm.nih.gov/pubmed/18167168

47. Kremers SPJ, Brug J, de Vries H, Engels RCME. Parenting style and adolescent fruit consumption. Appetite [Internet]. Department of Health Education and Health Promotion, Universiteit Maastricht, PO Box 616, 6200 MD Maastricht, The Netherlands. s.kremers@gvo.unimaas.nl SRC - Pubmed ID2 - 12880620 FG - 0; 2003;41:43–50. Available from: http://www.ncbi.nlm.nih.gov/sites/entrez?Db=pubmed&DbFrom=pubmed&Cmd=Link&LinkName=pubmed_pubmed&LinkReadableName=Related Articles&IdsFromResult=12880620&ordinalpos=3&itool=EntrezSystem2.PEntrez.Pubmed.Pubmed_ResultsPanel.Pubmed_RVDocSum

48. Kröller K, Warschburger P. Maternal feeding strategies and child's food intake: Considering weight and demographic influences using structural equation modeling. Int. J. Behav. Nutr. Phys. Act. 2009/11/26 ed. 2009;6:78.

49. Lee H, Keller KL. Children who are pressured to eat at home consume fewer high-fat foods in laboratory test meals. J. Acad. Nutr. Diet. 2012/06/27 ed. 2012;112:271–5.

50. Lo K, Cheung C, Lee A, Tam WW, Keung V. Associations between parental feeding styles and childhood eating habits: A survey of Hong Kong pre-school children. PLoS One. 2015/05/01 ed. 2015;10:e0124753.

51. Lopez N V, Ayala GX, Corder K, Eisenberg CM, Zive MM, Wood C, et al. Parent support and parent-mediated behaviors are associated with children's sugary beverage consumption. J. Acad. Nutr. Diet. [Internet]. 2012;112:541–7. Available from: <Go to ISI>://WOS:000303175900012

52. Loth KA, MacLehose RF, Larson N, Berge JM, Neumark-Sztainer D. Food availability, modeling and restriction: How are these different aspects of the family eating environment related to adolescent dietary intake? Appetite. 2015/09/04 ed. 2016;96:80–6.

53. McGowan L, Croker H, Wardle J, Cooke LJ. Environmental and individual determinants of core and non-core food and drink intake in preschool-aged children in the United Kingdom. Eur. J. Clin. Nutr. 2012/01/19 ed. 2012;66:322–8.

54. Monge-Rojas R, Smith-Castro V, Colon-Ramos U, Garita-Arce C, Sánchez-López M, Chinnock A. Parental feeding styles and adolescents' healthy eating habits. Structure and correlates of a Costa Rican questionnaire. Appetite [Internet]. Netherlands: Elsevier Science; 2010;55:253–62. Available from: http://search.ebscohost.com/login.aspx?direct=true&db=psyh&AN=2010-15051-001&site=ehost-live

55. Østbye T, Krause KM, Stroo M, Lovelady CA, Evenson KR, Peterson BL, et al. Parent-focused change to prevent obesity in preschoolers: Results from the KAN-DO study. Prev. Med. An Int. J. Devoted to Pract. Theory [Internet]. Netherlands: Elsevier Science; 2012;55:188–95. Available from: http://search.ebscohost.com/login.aspx?direct=true&db=psyh&AN=2012-24714-008&site=ehost-live

56. Nickelson J, Roseman MG, Forthofer MS. Associations between parental limits, school vending machine purchases, and soft drink consumption among Kentucky middle school students. J. Nutr. Educ. Behav. [Internet]. Elsevier. 6277 Sea Harbor Drive, Orlando, FL 32887-4800.; 2010;42:115–22. Available from: http://search.proquest.com/docview/61799816?accountid=12665

57. McPhie S, Skouteris H, Fuller-Tyszkiewicz M, McCabe M, Ricciardelli LA, Milgrom J, et al. Maternal predictors of preschool child-eating behaviours, food intake and body mass index: A prospective study. Early Child Dev. Care [Internet]. Routledge. , 325 Chestnut Street Suite 800, Philadelphia, PA 19106.; 2012;182:999–1014. Available from: http://search.proquest.com/docview/1314319147?accountid=12665

58. Melbye EL, Hansen H. Promotion and prevention focused feeding strategies: Exploring the effects on healthy and unhealthy child eating. Biomed Res. Int. [Internet]. 2015; Available from: <Go to ISI>://WOS:000360722800001

59. Melbye EL, Øgaard T, Øverby NC. Associations between parental feeding practices and child vegetable consumption. Mediation by child cognitions? Appetite [Internet]. Netherlands: Elsevier Science; 2013;69:23–30. Available from: http://search.ebscohost.com/login.aspx?direct=true&db=psyh&AN=2013-26519-

005&site=ehost-live

60. Melbye EL, Overby NC, Ogaard T. Child consumption of fruit and vegetables: The roles of child cognitions and parental feeding practices. Public Health Nutr. [Internet]. 2012;15:1047–55. Available from: <Go to ISI>://WOS:000304742200014

61. Palfreyman Z, Haycraft E, Meyer C. Development of the Parental Modelling of Eating Behaviours Scale (PARM): links with food intake among children and their mothers. Matern. Child Nutr. [Internet]. 2014;10:617–29. Available from: <Go to ISI>://WOS:000342749700013

62. Papaioannou MA, Cross MB, Power TG, Liu Y, Qu H, Shewchuk RM, et al. Feeding style differences in food parenting practices sssociated with fruit and vegetable intake in children from low-income families. J. Nutr. Educ. Behav. [Internet]. 2013;45:643–51. Available from: <Go to ISI>://WOS:000326596800022

63. Patrick H, Nicklas TA, Hughes SO, Morales M. The benefits of authoritative feeding style: caregiver feeding styles and children’s food consumption patterns. Appetite [Internet]. 2005;44:243–9. Available from: http://linkinghub.elsevier.com/retrieve/pii/S0195666304001448

64. Pearson N, Atkin AJ, Biddle SJ, Gorely T, Edwardson C. Parenting styles, family structure and adolescent dietary behaviour. Public Heal. Nutr. 2009/12/04 ed. 2010;13:1245–53.

65. Peters J, Sinn N, Campbell K, Lynch J. Parental influences on the diets of 2–5-year-old children: Systematic review of interventions. Early Child Dev. Care [Internet]. United Kingdom: Taylor & Francis; 2012;182:837–57. Available from: http://search.ebscohost.com/login.aspx?direct=true&db=psyh&AN=2012-16686-003&site=ehost-live

66. Ray C, Kalland M, Lehto R, Roos E. Does parental warmth and responsiveness moderate the associations between parenting practices and children’s health-related behaviors? J. Nutr. Educ. Behav. [Internet]. 2013;45:602–10. Available from: <Go to ISI>://WOS:000326596800017

67. Ray C, Roos E, Brug J, Behrendt I, Ehrenblad B, Yngve A, et al. Role of free school lunch in the associations between family-environmental factors and children’s fruit and vegetable intake in four European countries. Public Health Nutr. 2012/09/15 ed. 2013;16:1109–17.

68. Reinaerts E, de Nooijer J, Candel M, de Vries NK. Explaining school children’s fruit and vegetable consumption: The contributions of availability, accessibility, exposure, parental consumption and habit in addition to psychosocial factors. Appetite. 2006/11/18 ed. 2007;48:248–58.

69. Rodenburg G, Kremers SPJ, Oenema A, van de Mheen D. Associations of parental feeding styles with child snacking behaviour and weight in the context of general parenting. Public Health Nutr. 2013/03/27 ed. 2014;17:960–9.

70. Rodenburg G, Oenema A, Kremers SPJ, van de Mheen D. Parental and child fruit consumption in the context of general parenting, parental education and ethnic background. Appetite. 2011/11/19 ed. 2012;58:364–72.

71. Schwartz MB, Gilstad-Hayden K, Henderson KE, Luedicke J, Carroll-Scott A, Peters

SM, et al. The relationship between parental behaviors and children's sugary drink consumption is moderated by a television in the child's bedroom. Child. Obes. [Internet]. 2015;11:560–8. Available from: <Go to ISI>://WOS:000362269500010

72. Sdrali D, Anisiadou M, Goussia-Rizou M, Costarelli V. Adolescents' soft drinks consumption in family environment: A case study in Northern Greece. Int. J. Consum. Stud. [Internet]. 2010;34:684–90. Available from: <Go to ISI>://WOS:000282872000008

73. Sleddens EFC, Kremers SPJ, de Vries NK, Thijs C. Relationship between parental feeding styles and eating behaviours of Dutch children aged 6-7. Appetite. 2009/09/15 ed. 2010;54:30–6.

74. Sleddens EFC, Kremers SPJ, Stafleu A, Dagnelie PC, de Vries NK, Thijs C. Food parenting practices and child dietary behavior. Prospective relations and the moderating role of general parenting. Appetite. 2014/04/15 ed. 2014;79:42–50.

75. van Ansem WJC, van Lenthe FJ, Schrijvers CTM, Rodenburg G, van de Mheen D. Socio-economic inequalities in children's snack consumption and sugar-sweetened beverage consumption: The contribution of home environmental factors. Br. J. Nutr. [Internet]. 2014;112:467–76. Available from: <Go to ISI>://WOS:000339054100019

76. van Grieken A, Renders CM, van de Gaar VM, Hirasing RA, Raat H. Associations between the home environment and children's sweet beverage consumption at 2-year follow-up: the "Be active, eat right" study. Pediatr. Obes. [Internet]. 2015;10:126–33. Available from: <Go to ISI>://WOS:000351432400008

77. Van Lippevelde W, te Velde SJ, Verloigne M, De Bourdeaudhuij I, Manios Y, Bere E, et al. Associations between home- and family-related factors and fruit juice and soft drink intake among 10- to 12-year old children. The ENERGY project. Appetite. 2012/11/17 ed. 2013;61:59–65.

78. Taylor A, Wilson C, Slater A, Mohr P. Parent- and child-reported parenting. Associations with child weight-related outcomes. Appetite [Internet]. 2011;57:700–6. Available from: <Go to ISI>://WOS:000298128200020

79. van der Horst K, Kremers SPJ, Ferreira I, Singh A, Oenema A, Brug J. Perceived parenting style and practices and the consumption of sugar-sweetened beverages by adolescents. Health Educ. Res. [Internet]. 2007;22:295–304. Available from: http://www.her.oxfordjournals.org/cgi/doi/10.1093/her/cyl080

80. Van Strien T, van Niekerk R, Ouwens MA. Perceived parental food controlling practices are related to obesogenic or leptogenic child life style behaviors. Appetite [Internet]. 2009;53:151–4. Available from: <Go to ISI>://WOS:000268604500024

81. Vereecken CA, Keukelier E, Maes L. Influence of mother's educational level on food parenting practices and food habits of young children. Appetite [Internet]. 2004;43:93–103. Available from: <Go to ISI>://WOS:000223270500013

82. Wardle J, Carnell S, Cooke L. Parental control over feeding and children's fruit and vegetable intake: how are they related? J. Am. Diet. Assoc. 2005/01/26 ed. 2005;105:227–32.

83. Vereecken CA, Haerens L, De Bourdeaudhuij I, Maes L. The relationship between children's home food environment and dietary patterns in childhood and adolescence. Public Health Nutr. [Internet]. 2010;13:1729–35. Available from: <Go to ISI>://WOS:000284046000010

84. Vereecken CA, Legiest E, De Bourdeaudhuij I, Maes L. Associations between general parenting styles and specific food-related parenting practices and children's food consumption. Am. J. Heal. Promot. [Internet]. 2009;23:233–40. Available from: <Go to ISI>://WOS:000264217700003

85. Vereecken CA, Rovner A, Maes L. Associations of parenting styles, parental feeding practices and child characteristics with young children's fruit and vegetable consumption. Appetite [Internet]. 2010;55:589–96. Available from: <Go to ISI>://WOS:000285988900031

86. Wyse R, Campbell E, Nathan N, Wolfenden L. Associations between characteristics of the home food environment and fruit and vegetable intake in preschool children: A cross-sectional study. BMC Public Health. 2011/12/20 ed. 2011;11:938.

87. Xu H, Wen LM, Rissel C, Flood VM, Baur LA. Parenting style and dietary behaviour of young children. Findings from the Healthy Beginnings Trial. Appetite [Internet]. 2013;71:171–7. Available from: <Go to ISI>://WOS:000328868500022

88. Young EM, Fors SW, Hayes DM. Associations between perceived parent behaviors and middle school student fruit and vegetable consumption. J. Nutr. Educ. Behav. [Internet]. 2004;36:2–12. Available from: <Go to ISI>://WOS:000188662800003

89. Zahra J, Ford T, Jodrell D. Cross sectional survey of daily junk food consumption, irregular eating, mental and physical health and parenting style of British secondary school children. Child Care Heal. Dev [Internet]. United Kingdom: Wiley-Blackwell Publishing Ltd.; 2014;40:481–91. Available from: http://search.ebscohost.com/login.aspx?direct=true&db=psyh&AN=2014-23768-004&site=ehost-live
